# Eumalacostracan phylogeny and total evidence: limitations of the usual suspects

**DOI:** 10.1186/1471-2148-9-21

**Published:** 2009-01-27

**Authors:** Ronald A Jenner, Ciara Ní Dhubhghaill, Matteo P Ferla, Matthew A Wills

**Affiliations:** 1 Department of Biology and Biochemistry, University of Bath, The Avenue, Claverton Down, Bath, BA2 7AY, UK

## Abstract

**Background:**

The phylogeny of Eumalacostraca (Crustacea) remains elusive, despite over a century of interest. Recent morphological and molecular phylogenies appear highly incongruent, but this has not been assessed quantitatively. Moreover, 18S rRNA trees show striking branch length differences between species, accompanied by a conspicuous clustering of taxa with similar branch lengths. Surprisingly, previous research found no rate heterogeneity. Hitherto, no phylogenetic analysis of all major eumalacostracan taxa (orders) has either combined evidence from multiple loci, or combined molecular and morphological evidence.

**Results:**

We combined evidence from four nuclear ribosomal and mitochondrial loci (18S rRNA, 28S rRNA, 16S rRNA, and cytochrome *c *oxidase subunit I) with a newly synthesized morphological dataset. We tested the homogeneity of data partitions, both in terms of character congruence and the topological congruence of inferred trees. We also performed Bayesian and parsimony analyses on separate and combined partitions, and tested the contribution of each partition. We tested for potential long-branch attraction (LBA) using taxon deletion experiments, and with relative rate tests. Additionally we searched for molecular polytomies (spurious clades). Lastly, we investigated the phylogenetic stability of taxa, and assessed their impact on inferred relationships over the whole tree. We detected significant conflict between data partitions, especially between morphology and molecules. We found significant rate heterogeneity between species for both the 18S rRNA and combined datasets, introducing the possibility of LBA. As a test case, we showed that LBA probably affected the position of Spelaeogriphacea in the combined molecular evidence analysis. We also demonstrated that several clades, including the previously reported and surprising clade of Amphipoda plus Spelaeogriphacea, are 'supported' by zero length branches. Furthermore we showed that different sets of taxa have the greatest impact upon the relationships within molecular versus morphological trees.

**Conclusion:**

Rate heterogeneity and conflict between data partitions mean that existing molecular and morphological evidence is unable to resolve a well-supported eumalacostracan phylogeny. We believe that it will be necessary to look beyond the most commonly utilized sources of data (nuclear ribosomal and mitochondrial sequences) to obtain a robust tree in the future.

## Background

Attempts to infer the phylogeny of eumalacostracans have been high on the agenda of systematic zoology at least " [s]ince the awakening in natural science which followed the publication of the Origin of species" [1]. This is unsurprising, firstly because several of the most influential zoologists of the late 19^th ^and early 20^th ^century were carcinologists, and secondly because the erstwhile 'higher Crustacea' houses the majority of economically and commercially important species of edible crabs, shrimps, and lobsters. What *is *surprising, however, is that in the 21^st ^century, when considerable resources are being directed towards "assembling the tree of life", no major initiative is focused on resolving relationships within this, the most diverse of all crustacean clades [2]. By contrast, relatively large programs are underway to tackle both broader (e.g., arthropod phylogeny: NSF DEB-0120635, awarded to C. Cunningham, J. Martin, J. Regier, J. Thorne, and J. Shultz) and more narrow (e.g., decapod relationships; NSF awards DEB-EF-0531603 (F. Felder), DEB-EF-0531616 (J. Martin), DEB-EF-0531670 (R. Feldmann and C. Schweitzer) and DEB-EF-0531762 (K. A. Crandall and N. Hanegan)) phylogenetic problems. A concerted effort to resolve eumalacostracan phylogeny would complement these efforts, providing a valuable supplement and broader interpretative framework, respectively.

The ongoing development of faster and cheaper DNA sequencing techniques, coupled with advances in analytical methods, are encouraging researchers to revisit old and recalcitrant phylogenetic problems. Before embarking on such a revision for the Malacostraca, it is therefore extremely timely to take stock of the present state of knowledge by synthesizing and analyzing all of the presently available data. This is necessary for several reasons.

Firstly, there is still no robust consensus on malacostracan phylogeny, despite recent and comprehensive analyses of morphological and molecular data [3-6]. Although there is some congruence between the latest morphological analyses, some striking incongruities are present as well [3,4]. Here, we discuss critically some of the most striking unresolved issues, and integrate previously published morphological data sets [3,4,7-9] into a new matrix.

Secondly, molecular approaches to eumalacostracan relationships are still in their infancy. Although sequence data have been applied to a variety of taxonomically restricted questions [10,11], only two studies were based on sufficient taxon sampling to be able to focus on resolving the major relationships between the traditional higher-level malacostracan taxa [5,6]. In addition, several studies of deeper arthropod phylogeny [12-15] have included small numbers of representative eumalacostracans, but taxon sampling is too sparse to interpret these results straightforwardly. The two comprehensive studies aimed explicitly at resolving eumalacostracan phylogeny are based on 18S rRNA [5,6]. However, potential problems with these analyses, such as long-branch attraction, and the availability of new sequences of 28S rRNA, 16S rRNA, and cytochrome *c *oxidase subunit I for previously unsampled taxa, make a multilocus re-evaluation of eumalacostracan phylogeny opportune.

Thirdly, a comparison of the morphological and molecular phylogenies of Eumalacostraca reveals a number of "puzzling" [5] or even "disturbing" [3] conflicts that have so far evaded satisfactory explanation, or testing in a total evidence framework. These conflicts are easily revealed by a topological comparison of the molecular and morphological cladograms, but we perform the first quantitative test of whether morphology and molecules present significantly different signals.

The two most recent and most comprehensive morphological phylogenetic analyses of eumalacostracan phylogeny are Richter & Scholtz (2001) [4] and Poore (2005) [3]. These studies evaluated and synthesized previous evidence for malacostracan phylogeny, and agree on the following:

• Peracarida including Thermosbaenacea (= Pancarida) is monophyletic

• Mysidacea is monophyletic

• Mictacea and Spelaeogriphacea are sister taxa

In contrast, these studies disagree about the positions of Decapoda, Euphausiacea, Mysidacea, Cumacea, Tanaidacea, and Isopoda. However, because Poore (2005) [3] focused on resolving peracarid relationships while Richter & Scholtz (2001) [4] had the broader remit of malacostracan phylogeny, these studies are not strictly comparable.

Poore (2005: 2) [3] stated that his morphological data set was "essentially a compilation of those [morphological characters] used previously but with additions", which leads to the reasonable expectation that Poore's analysis should be the most severe morphological test of peracarid (and possibly wider eumalacostracan) phylogeny published to date. We note, however, that Poore chose not to incorporate several characters from Richter & Scholtz (2001) [4], including some that he conceded were "the most significant" to bear on certain relationships within the wider Eumalacostraca. These were excluded, quite reasonably, because they did not contribute specifically to resolving peracarid relationships. All have been reinstated in the present analyses.

We synthesized the character sets of Richter & Scholtz (2001) and Poore (2005) [3,4] with those of previous works [4,8,9,16] as well as newly published information to derive a revised morphological hypothesis of malacostracan phylogeny.

Published molecular phylogenies focusing expressly on eumalacostracan relationships are derived from 28S rRNA and 18S rRNA sequences [5,6,10,15]. Congruence between these is limited, partly because of differences in taxon sampling, but also (as we show here), because the two molecules contain conflicting signals that are not strong enough to resolve relationships at all levels. However, 28S rRNA and 18S rRNA do agree that:

• Mysids are more closely related to euphausiaceans and stomatopods than to the other peracarids

• Isopods and amphipods are *not *sister taxa

• Decapods, euphausiaceans and stomatopods may be part of a clade separate from the peracarids

The phylogenetic positions of all other taxa are highly variable. Different analytical methods yield different trees [5,15], all of which have very low levels of clade support.

One striking aspect of the 18S rRNA trees in Spears et al. (2005) [5] and Meland & Willassen (2007) [6] is the large difference in branch lengths. All peracarid branches, (with the exception of those of Mysida), appear to be significantly longer than those of the non-peracarid malacostracans. Particularly noteworthy is the clade of Amphipoda and Spelaeogriphacea, which is supported by both studies, but which lacks any known morphological support [3,5]. This anomalous clade groups the most divergent sequences included in these studies, leading us to suspect long-branch attraction (LBA). Although Meland & Willassen (2007) [6] did not discuss the possibility of LBA, Spears et al. (2005) [5] dismissed it. Our reinvestigation suggests that LBA may, in fact, be a significant problem.

The most striking result of the recent literature is the apparent conflict between morphology [3,4,8,9,16-18] and molecules [5,6,10,11]. This implies significant homoplasy in either molecular or morphological evidence (or both). The only sister group relationship to receive independent support from molecules and morphology is that between Euphausiacea and Decapoda (Eucarida). Even so, this clade is contradicted by several other morphological and molecular phylogenetic analyses. Considering clade membership rather than sister groupings admits a few more areas of agreement. For example, some studies present molecular and morphological support for a clade comprising Cumacea, Isopoda, and Tanaidacea.

The most striking differences between molecular (principally 18S rRNA) and morphological (principally Richter & Scholtz 2001 and Poore 2005 [3,4]) trees, respectively, are the following:

• Monophyly vs. polyphyly of Mysidacea

• Monophyly vs. polyphyly of Peracarida (through exclusion of Mysida from Peracarida)

• Absence vs. presence of a clade minimally including Stomatopoda, Euphausiacea and Mysida

• Absence vs. presence of a clade minimally including Amphipoda, Spelaeogriphacea and Lophogastrida

In this paper we combine, for the first time, molecular data from four nuclear and mitochondrial loci (18S rRNA, 28S rRNA, 16S rRNA, cytochrome *c *oxidase subunit I) along with morphological evidence for higher-level eumalacostracan relationships. Among other things, this represents the first test of the phylogenetic position of Bathynellacea and the monophyly of Syncarida using combined molecular evidence (16S rRNA and cytochrome *c *oxidase subunit I) [11,19]. The combined discussions and results presented in this paper should be valuable as a guide to any future phylogenetic analysis of this diverse clade. They reveal the limitations of published evidence, and highlight where understanding is lacking.

## Methods

### Morphology

We synthesized a new morphological cladistic dataset by integrating previous matrices (additional files 1, 2) [3,4,7-9,16]. The data sets of Wills (1997, 1998) [7,9] and Schram and Hof (1998) [16] were originally compiled to address wider questions of crustacean phylogeny. In removing most non-malacostracan taxa (with the exception of an outgroup comprising Leptostraca, Anostraca, Notostraca and Brachypoda) a number of characters were rendered uninformative for the residual taxon sample. Other additive (or "ordered") characters had "intermediate" states removed, and were therefore recoded to reflect only those states present in the remaining sample. Poore's (2005) [3] data set contained a restricted sample of non-peracarid malacostracans, such that additional taxa were coded for some characters. Groups represented in Poore (2005) [3] by more than one OTU (Mictacea and Spelaeogriphacea) were recoded as polymorphic taxa. Pires (1987) [8] did not present a matrix as such, and most characters were subsumed within those of later authors. More generally, characters represented in two or more matrices were coded to reflect the most recent study. We have typically coded limited uncertainty and polymorphic states rather than introducing assumptions regarding the groundplans for our terminals. Characters relating to numbers of podomeres have generally been coded to reflect all of the variation between orders. Many crustacean orders contain exemplars in which given rami for given appendages may be either reduced (one or two podomeres) or absent altogether. For this reason, we have predominantly included "zero podomeres" as a state within characters coding for podomere numbers. The alternative would be to introduce an additional character for the presence or absence of a given ramus, with "podomere" characters coded as inapplicable for terminals lacking the ramus. Possible ordering and weighting schemes for such characters have been discussed elsewhere in detail [9], and similar principles have been applied here. In some analyses, therefore, characters relating to numbers of limb elements (podomeres, endites, etc.) and numbers of somites have been ordered, while those relating to numbers of limb elements have also been scaled to unit weight.

### Molecules

Spears et al. (2005) and Meland & Willassen (2007) [5,6] have published the most comprehensively sampled molecular phylogenies of the higher-level taxa within Malacostraca (14 of the 15 recognised, excluding Bathynellacea). Both analyses are based on 18S rRNA. In contrast, Jarman et al. (2000) [10] included just 10 of the 15 recognized higher-level taxa in the first phylogenetic analysis of Malacostraca based on 28S rRNA. Subsequent more inclusive analyses of wider arthropod relationships have generally included a more restricted sample of malacostracan higher-level taxa [12-15].

### Taxon selection

Our choice of taxa was dictated by several considerations. Firstly, we started with the aligned 18S rRNA dataset of Meland & Willassen (2007) [6] facilitating a direct comparison with this study and that of Spears et al. (2005) [5] (additional file 3).

Secondly, we concatenated the data partitions for 18S rRNA, 28S rRNA, 16S rRNA, cytochrome *c *oxidase subunit I, and morphology (additional files 4, 5, 6, 7, 8). In order to maximize data density per taxon we created composite (chimerical) higher-level terminals for several taxa (see Table 1), which is a reasonable strategy in multilocus and phylogenomic analyses [20,21]. For composites, we included the most closely related species available, using generic (or higher when necessary) membership as proxies for relatedness. This strategy should not distort phylogenetic analyses, provided the composite taxa are certifiably monophyletic with respect to the others sampled [22]. This is well supported for the terminals used here [4]. We acknowledge that this strategy precludes testing explicitly the validity of our assumed monophyla. Some authors therefore prefer not to amalgamate, despite the introduction of large amounts of "missing data" [23]. Although missing data can reduce consensus resolution, it does not necessarily yield spurious relationships [24].

**Table 1:** GenBank (NCBI) accession numbers and composite terminal taxa.

**Taxon**	**Species**	**18S rRNA**	**28S rRNA**	**16S rRNA**	**Cyt. *c *ox. sub. I**
Leptostraca	*Nebalia sp*.	L81945			
	*Dahlella caldariensis*				U92670
	*Paranebalia longipes*		EF189655	AY744909	
Stomatopoda	*Squilla empusa*	L81946^1^	AY210842^1^	AF107617^1^	DQ191684^1^
	*Gonodactylus sp*.	L81947^2^			
	*Gonodactylus graphurus*			Af133678^2^	AF048822^2^
	*Gonodactylus viridus*		AY739190^2^		
Decapoda	*Callinectes sapidus*	AY781436^1^	AY739194^1^	CSU75267^1^	AY682072^1^
	*Panulirus argus*	AY781435^2^	AY210833^2^	AF502947^2^	AF339452^2^
	*Homarus americanus*	AF235971^3^	DQ079788^3^	DQ666843^3^	DQ889104^3^
Euphausiacea	*Meganyctiphanes norvegica*	AY781434^1^	AY744900^1^	AY744910^1^	AF177191^1^
	*Nyctiphanes simplex*	AY781433^2^		AY574929^2^	AY601092^2^
Bathynellacea	*Atopobathynella wattsi*				EU350222
	*Iberobathynella magna*			AF5032570	
Anaspidacea	*Anaspides tasmaniae*	L81948	AF169720	AF133694	DQ889076
Thermosbaenacea	*Thethysbaena argentarii*	AY781415	DQ470654	DQ470612	
Lophogastrida	*Neognathophausia ingens*	AY781416	AF244095		DQ889115
Mysida	*Neomysis integer*	AY781420^1^			
	*Mysis segerstralei*		EU233536^1^	DQ189201^1^	EF609275^1^
	*Hemimysis abyssicola*	AM422508^2^			
	*Hemimysis margalefi*				AM114209^2^
	*Hemimysis anomala*		EU233527^2^		
Amphipoda	*Gammarus oceanicus*	AY781422^1^		AY926728^1^	AY926674^1^
	*Gammarus lichuanensis*		EF583002^1^		EF570357^3^
	*Phronima sp*.	AY781424^2^			
	*Phronima bucepahala*				EF989680^2^
	*Primno macropa*		EU375505^2^		
Isopoda	*Asellus racovitzai*	AY781426^1^			
	*Asellus aquaticus*		DQ144749^1^	DQ305106^1^	DQ144795^1^
	*Idotea metallica*	AY781427^2^			AF241928^2^
	*Idotea baltica*		AY739187^2^		
	*Idotea resecata*			AF259538^2^	
	*Paramphisopus palustris*	AY781425^3^		AF259533^3^	EF203022^3^
	*Colubotelson sp*.		AF169711^3^		
Cumacea	*Diastylis sculpta*	AY781431^1^		U81512^1^	AF137510^1^
	*Spilocuma salomani*	AY781432^2^			
	*Mancocuma stellifera*				AF137520^2^
	*Cumopsis fagei*			AJ388111^2^	
	*Cyclapsis caprella*		AF169712^2^		
	*Eudorella pusilla*				AF137516^3^
Tanaidacea	*Tanais dulongi*	AY781428			
	*Tanaidacea sp*.				AF520452
	*Apseudes latreillei*			AJ388110	
	*Paratanais sp*.		AF169710		
Mictacea	*Thetispelecaris remex*	AY781421			
Spelaeogriphacea	*Spelaeogriphus lepidops*	AY781414			

For several analyses some higher taxa have two or more representatives (e.g., Stomatopoda 1 and Stomatopoda 2, etc.). The superscripted numerals in the table indicate to which (composite) taxa the sequences have contributed (e.g. Cumacea1 is based on the sequences with numeral '1'). The analyses based on combined molecular and morphological evidence only include the sequences with numeral '1'.

Thirdly, for taxa with multiple representative species we excluded most of those with data for just one or two loci. This explains why we sometimes included fewer representatives of certain groups (e.g., Tanaidacea, Mysida, Lophogastrida, and Decapoda) than Meland & Willassen (2007) [6]. The problematic and rarely-sampled orders Mictacea and Spelaeogriphacea were included on the basis of 18S rRNA data alone, while Bathynellacea was represented by 16S rRNA and cytochrome *c *oxidase subunit I data. *Stygiomysis *was excluded. Again, we note that the inclusion of missing data need not obfuscate or distort inferred relationships [24]. Moreover, missing data is not the only thing with the potential to influence trees: small differences in taxon or character sampling can have radical effects [25]. Hence, there are two issues. The first is whether the removal of taxa that share few characters with the majority of the others will result in a different tree. The second is whether these same taxa are themselves resolved in a misleading position. The first issue was addressed, at least in part, by first order jackknifing. This would demonstrate that both Mictacea and Bathynellacea could be removed from the parsimony analysis, with no effect upon the relationships of the remaining terminals. Removal of the Spelaeogriphacea had a small, localized effect, with no implications for the positions of Mictacea or Bathynellacea. We also investigated this in the Bayesian analyses, with a similar outcome. Removal of Spelaeogriphacea left the topology of the combined molecular analysis unchanged, while removal of Bathynellacea only affected the position of the Mictacea (shifting it down the tree by two nodes that lacked significant support). The second issue – testing the placement of these taxa – can only be addressed by collecting more data: filling in the gaps or sequencing new genes. However, this is true for any phylogenetic hypothesis, irrespective of putative missing data problems.

Fourthly, we reduced the number of species in the combined molecular and morphological evidence analyses so that each OTU was represented by a single taxon. This was done to prevent the results from being affected by the replicated morphological ground patterns, which would strongly bias the analyses in favour of the monophyly of the higher taxa with multiple representative species.

For the molecular and combined evidence analyses we designated Leptostraca as the outgroup. There is general consensus in the malacostracan literature that leptostracans are the sister group to the remaining malacostracans, and the monophyly of Eumalacostraca is well supported by morphology [4]. This is also supported by some larger scale molecular and combined evidence analyses [13,15,26].

### Data partitions and alignments

All sequences (except 18S rRNA sequences, which were kindly provided by K. Meland), were downloaded from the GenBank, National Center for Biotechnology Information (NCBI) (see Table 1 for accession numbers), and with the exception of the 18S rRNA data, were aligned online with T-coffee . For the 18S rRNA data we used the alignment of Meland & Willassen (2007) [6], which incorporates secondary structure information. Ambiguously aligned regions were determined by the program Gblocks version 0.91 b [27], and excluded from the analyses. After trying a variety of settings, the final Gblocks settings were selected to yield a good quality alignment while not sacrificing an unnecessarily large amount of data. Nevertheless, ambiguously aligned regions and especially pronounced length variation between species in the ribosomal genes necessitated the removal of 49%, 65%, and 85% of the 18S, 16S, and 28S alignments, respectively. The settings were the following for the 18S rRNA, 16S rRNA and 28S rRNA partitions respectively: [1: 27; 2: 44; 3: 8; 4: 4; 5: all]; [1: 11; 2: 11; 3: 8; 4: 5; 5: with half]; [1: 10; 2: 10; 3: 8; 4: 5; 5: with half]. The cytochrome *c *oxidase subunit I partition did not contain any ambiguously aligned regions, and the alignment was checked with respect to the amino acid alignment.

The partitions and the character exclusion sets based on Gblocks are as follows (positions continuous in the concatenated dataset):

### Partitions

18S rRNA: 1-3249

28S rRNA: 3250-6590

cytochrome *c *oxidase subunit I: 6591-7205

16S rRNA: 7206-8316

morphology: 8317-8493

### Character exclusion sets

18S rRNA: 82-140 303-361 403-420 547-810 1125-1169 1379-1409 1422-1466 1514-1589 1636-1719 1737-2446 2824-2834 2881-2955 2986-3043 3099-3126 3175-3186

28S rRNA: 3250-4590 4621 4646-4648 4660 4667-4712 4723-4754 4763-5116 5134-5145 5155 5156 5168-5172 5178-5183 5227-5234 5246 5247 5279-5281 5303-5310 5335-5345 5369-5579 5598 5599 5615 5624 5625 5680-5691 5703-5716 5742-5745 5767 5768 5781-5783 5812-5815 5825 5835-5838 5876-6590

16S rRNA: 7206-7225 7233-7251 7263-7293 7299-7455 7481 7488 7489 7511-7514 7521 7530 7542-7549 7557-7569 7577-7584 7657-7687 7698-7704 7712-7724 7734 7735 7743-7747 7758-7764 7775-7778 7785-7787 7803-7821 7834-7840 7848 7849 7859 7890 7913 7944-7948 7965 7966 7972-7979 7994-8316

The concatenated molecular data set includes a total of 3226 aligned positions, with 1674, 531, 406, and 615 positions for the 18S rRNA, 28S rRNA, 16S rRNA, and cytochrome *c *oxidase subunit I partitions, respectively.

### Phylogenetic signal and phylogenetic analyses

The data were analysed using both parsimony and Bayesian inference, using PAUP* [28] and MrBayes [29] respectively. We performed Bayesian and parsimony analyses on all separate partitions and the combined data.

For the parsimony analyses we performed heuristic searches consisting of 1,000 (or more where stated) random addition replicates with TBR branch swapping. All molecular characters were treated as unordered and equally weighted, offering a contrast with the Bayesian analyses (where complex models of molecular evolution were used). The ordering and weighting of morphological characters in the parsimony analyses is as defined in additional file 1. Bootstrapping analyses were based on 2,500 or more resamplings, each with 1,000 random additions and TBR swapping. For the morphological data set, bootstrapped trees were additionally used to determine maximum leaf stabilities (LS) [30-34] using RadCon [30]. In rooted trees, the leaf stability of a taxon is calculated as the average of the support values for all three-taxon statements including that taxon. Stable taxa will contribute to well-supported triplets. Taxa with lower leaf stabilities are more likely to impact negatively upon apparent support. Hence, leaf stabilities can be used to measure directly how far the relationships of a given terminal to all other terminals are supported, which in turn offers a proxy for the likely impact of a given taxon on global measures of tree support. We also used first order jackknifing to determine the impact upon relationships of removing individual taxa [25]. Reference trees were produced by pruning each taxon from the set of MPTs from the simultaneous analysis of all taxa. These were compared with trees resulting from additional parsimony analyses sequentially omitting each taxon from the outset. The impact upon apparent relationships was measured using two indices: the symmetric difference distance on full splits (RF of Robinson and Foulds 1981 [35]) and the maximum agreement subtree distance (d1 of Finden & Gordon 1985 [36]). The RF measures the difference between two trees as the number of nodes unique to both, while d1 reports the number of taxa missing from the maximum agreement subtree. These differ conceptually, and may differ markedly in practice. Where comparisons were between sets of trees, we calculated the mean distance between each tree in one set and the most similar tree in the other set (such that identical sets of trees have no difference). For the combined evidence analyses, we calculated partitioned Bremer support indices [37] using TreeRot version 3 [38].

For the Bayesian analyses we used MrModeltest [39] to determine the best-fitting model for each data partition, excluding ambiguously aligned regions from the calculations. This resulted in the following models being used for all analyses of separate or combined partitions: GTR + G + I (general time-reversible model + gamma distributed rates of substitution + estimated proportion of invariant sites) for 18S rRNA and 16S rRNA; GTR + G for 28S rRNA and cytochrome *c *oxidase subunit I. We did not partition stem and loop regions for the 18S rRNA and 28S rRNA genes. With respect to the 18S rRNA data, both Spears et al. (2005: 134) [5] and Meland & Willassen (2007: 1090) [6] note that Bayesian analyses with the stem and loop regions of the 18S rRNA molecule treated the same or as unlinked partitions resulted in "highly congruent" results. For the morphological partition, we used a common-mechanism maximum-likelihood model, with a gamma distribution of rates (Mkv+G model of Lewis, 2001 [40]). Unless stated otherwise we ran four chains, of which three were heated. We sampled every 200 generations, and used a 25% burn-in. In all combined analyses, we allowed rates to vary independently for each partition. For the combined molecular and morphological analyses all morphological characters were treated as equally weighted and non-additive. For individual runs, additional parameters were:

- 18S rRNA: seven million generations, average standard deviation of split frequencies: 0.007.

- 28S rRNA: 2039000 generations before automatic average standard deviation of split frequencies (0.01) was reached.

- 16S rRNA: three million generations, average standard deviation of split frequencies: 0.006.

- Cytochrome *c *oxidase subunit I: five million generations, average standard deviation of split frequencies: 0.008.

- Morphology (MOR): three million generations, average standard deviation of split frequencies: 0.003 (all non-additive characters)/0.002 (some characters treated as additive).

- Combined molecules (MOL): seven chains (six heated), sample and print frequency: 200, seven million generations, average standard deviation of split frequencies: 0.0094.

- MOL minus Spelaeogriphacea: seven chains (six heated), sample and print frequency: 200, four million generations, average standard deviation of split frequencies: 0.007.

- MOL minus Amphipoda: five chains (four heated), sample and print frequency: 200, six million generations, average standard deviation of split frequencies: 0.007.

- Combined molecules and morphology (MOLMOR): six chains (five heated), six million generations, average standard deviation of split frequencies: 0.004.

- MOLMOR minus Spelaeogriphacea: six chains (five heated), sample and print frequency: 400, six million generations, average standard deviation of split frequencies: 0.003.

- MOLMOR minus Amphipoda: six chains (five heated), sample and print frequency: 400, six million generations, average standard deviation of split frequencies: 0.008.

In the combined Bayesian analyses we unlinked the parameters for priors on substitution rates (revmatpr), stationary nucleotide frequencies (statefreqpr), shape of the gamma distribution of rate variation (shapepr), and proportion of invariant sites (pinvarpr) for all molecular data partitions.

For the ILD test [41] we made all possible comparisons of individual loci (Table 2) and between these and morphology (Table 3) (in addition to a single test of all partitions analysed simultaneously). For each comparison, we removed all taxa present in only one partition. Hence, the number of taxa analysed was not uniform. This necessitated the further removal of uninformative characters from one or both partitions so that the number of characters contributed by an individual partition was also variable. Each concatenated data set (comprising two loci, or one locus and morphology) was analysed in PAUP* with 1,000 random partitions ("hompart"). All characters were treated as unordered and unweighted for simplicity. Heuristic searches were used throughout, with 1,000 random additions of taxa followed by TBR branch swapping. For the molecular data set (Table 2), we also estimated the topological Mickevich-Farris ILD or TILD [42]. In this implementation, we inferred a strict consensus from each partition, recoded these using group inclusion characters (matrix representation in PAUP*), and subjected the resultant combined matrix to the ILD test. We note that the TILD test can be applied to partitions and trees comprising incompletely overlapping sets of taxa, but preferred to make a direct comparison with the ILD results here.

**Table 2:** Results of ILD and TILD tests for the concatenated data set of four molecules.

	**18S rRNA**	**28S rRNA**	**16S rRNA**	**Cyt. *c *ox. Sub. I**
**18S rRNA**		467/155	354/225	444/318
**28S rRNA**	0.004/na (19)		119/217	155/313
**16S rRNA**	0.001/0.002 (18)	0.001/0.007 (16)		222/308
**Cyt**. ***c *ox. sub. I**	0.001/0.002 (20)	0.001/0.001 (18)	0.035/0.001 (18)	

Above diagonal: Number of informative characters for row/column. Below diagonal: P value from ILD/TILD test (< 0.008 to reject homogeneity), with number of taxa in brackets. TILD tests were based on strict consensus trees (which was completely unresolved for 28S rRNA in its comparison with 18S rRNA).

**Table 3:** Results of the ILD tests for the concatenated total evidence data set (molecules and morphology).

	**18S rRNA**	**28S rRNA**	**16S rRNA**	**Cyt. *c *ox. sub. I**	**Morphology**
**18S rRNA**		273/100	277/184	281/273	443/128
**28S rRNA**	0.014 (11)		82/168	78/261	100/119
**16S rRNA**	0.001 (11)	0.001 (10)		282/194	194/124
**Cyt**. ***c *ox. sub. I**	0.001 (11)	0.001 (10)	0.012 (13)		282/126
**Morphology**	0.001 (14)	0.001 (10)	0.001 (12)	0.001 (12)	

Above diagonal: Number of informative characters for row/column. Below diagonal: P value from ILD test (< 0.005 to reject homogeneity), with number of taxa in brackets.

### Branch lengths, long-branch attraction and relative substitution rates

We assessed the possibility of long-branch attraction artefacts in our analyses with a series of exploratory analyses (there are no conclusive tests *per se*). Firstly, we performed a distance-based relative rate test with RRTree [43] on the 18S rRNA data to test whether taxa differed significantly in their relative substitution rates. We compared the results considering each taxon as a separate lineage, and using a pre-defined guide tree to allow rates to be compared between different supra-specific clades (using the 18S rRNA topology of Meland & Willassen 2007 [6]). This method has been used to identify fast-clock organisms for exclusion from phylogenetic analyses [44,45].

Secondly, we utilized likelihood ratio tests in PAUP* to determine whether the sequences evolved at similar rates. We did this by comparing the likelihoods of the trees both with and without a molecular clock enforced. The likelihood ratio was then calculated as 2(lnL1-lnL2), where L1 is the null hypothesis (clock assumed), which is a subset or special case (in nested models) of the alternative hypothesis L2 (no clock assumed). We assumed s-2 degrees of freedom, where n is the number of terminals. We used this test in addition to the above distance-based relative rate test for several reasons. Spears et al. (2005) [5] rejected the possibility of LBA in their 18S rRNA tree based on the basis of a likelihood ratio test, so we re-analysed our data in the same way. More importantly, distance-based relative rate tests and likelihood ratio tests may differ in their sensitivity [46], so we applied both here. More prosaically, and despite repeated attempts, we failed to prevent RRTree from crashing when analyzing the concatenated molecular data (possibly a function of the amount of sequence data the program is able to handle). Consequently, we resorted to likelihood ratio testing for the concatenated molecular data.

Thirdly, we performed taxon exclusion experiments designed to test whether taxa with high substitution rates are artificially attracted to each other [47]. If LBA occurs by attraction of two long-branch taxa, removal of one of these from the analysis may allow the other taxon to find its proper place in the phylogeny. If the remaining taxon jumps to a different position in the tree, then it is possible that the initial clade was a LBA artefact.

Fourthly, we evaluated concordance between the analyses of the separate data partitions, and between the parsimony and Bayesian analyses. Both methodological discordance of results (based on the fact that different methods differ in their ability to prevent LBA), and the lack of morphological support for a molecular clade (an admittedly weak criterion) have been taken as possible indications of LBA in the literature [47].

Using PAUP *, we performed a likelihood ratio test of internal branch lengths for the 18S rRNA sequences and the combined molecular evidence. This allowed us to test whether the very short internal branches were significantly different from zero length (an option available under "likelihood settings").

## Results

**Figure 1:**
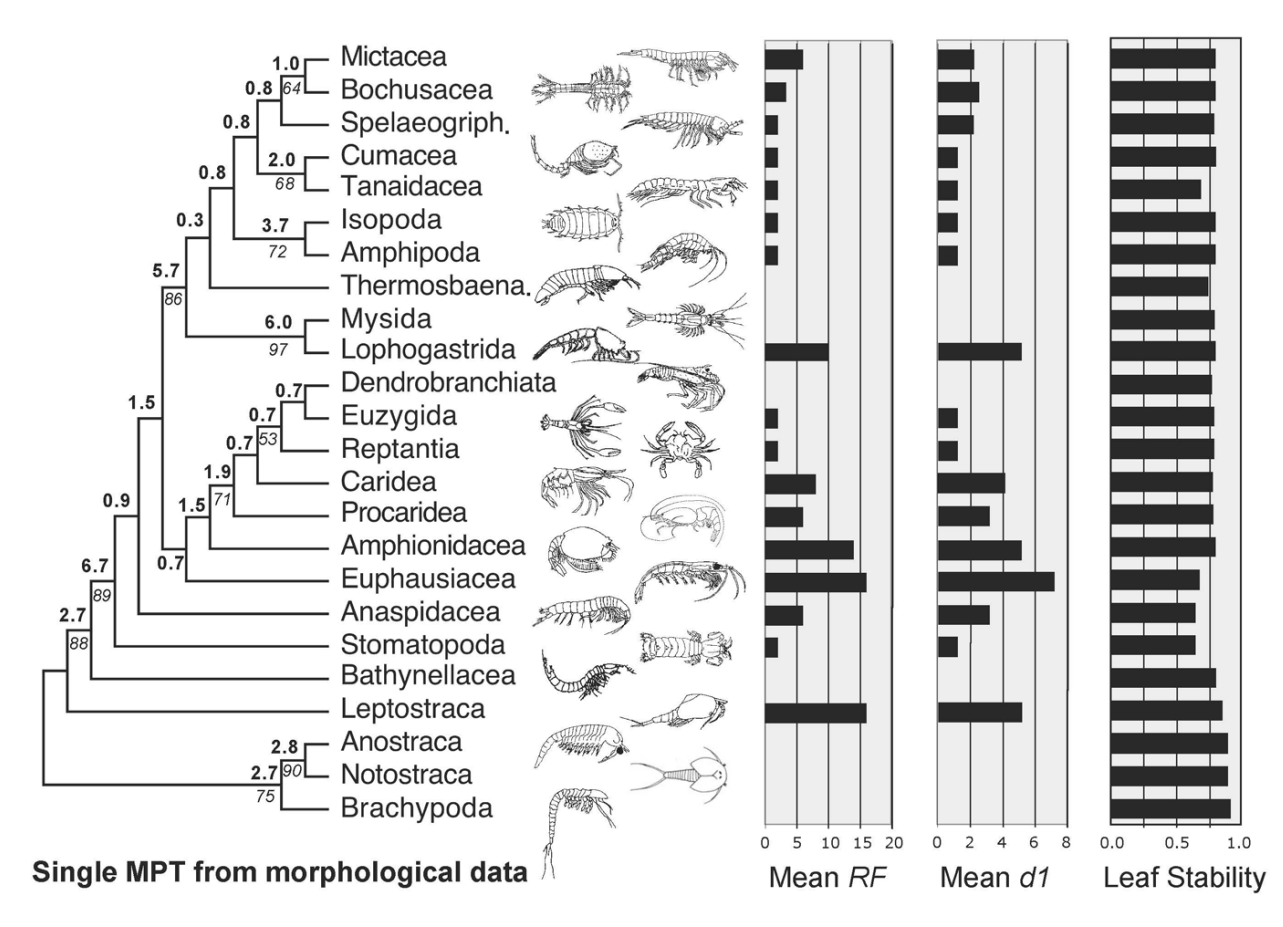
**Phylogeny of Eumalacostraca based on parsimony analysis of 177 morphological characters (CI = 0.42, RI = 0.62)**. Data set compiled principally from the work of Pires (1987), Wills (1997, 1998), Schram & Hof (1998), Richter & Scholtz (2001) and Poore (2005) [3,4,7-9,16]. Characters relating to numbers of somites, limbs and limb elements (podomeres and endites) have been ordered. Characters relating to limb elements have also been scaled to unity (ranged). Bold figures on branches indicate Bremer support. Italic figures are bootstrap percentages where these exceed 50% (10,000 equiprobable character resamplings, each with 1,000 random additions and TBR). Histograms of mean RF and mean d1 relate respectively to the symmetrical difference distance and maximum agreement subtree distance measures of the impact upon relationships of removing individual taxa (first order jackknife). Leaf stability is calculated as the maximum value based on trees from the first 2,000 bootstrap replicates.

**Figure 2:**
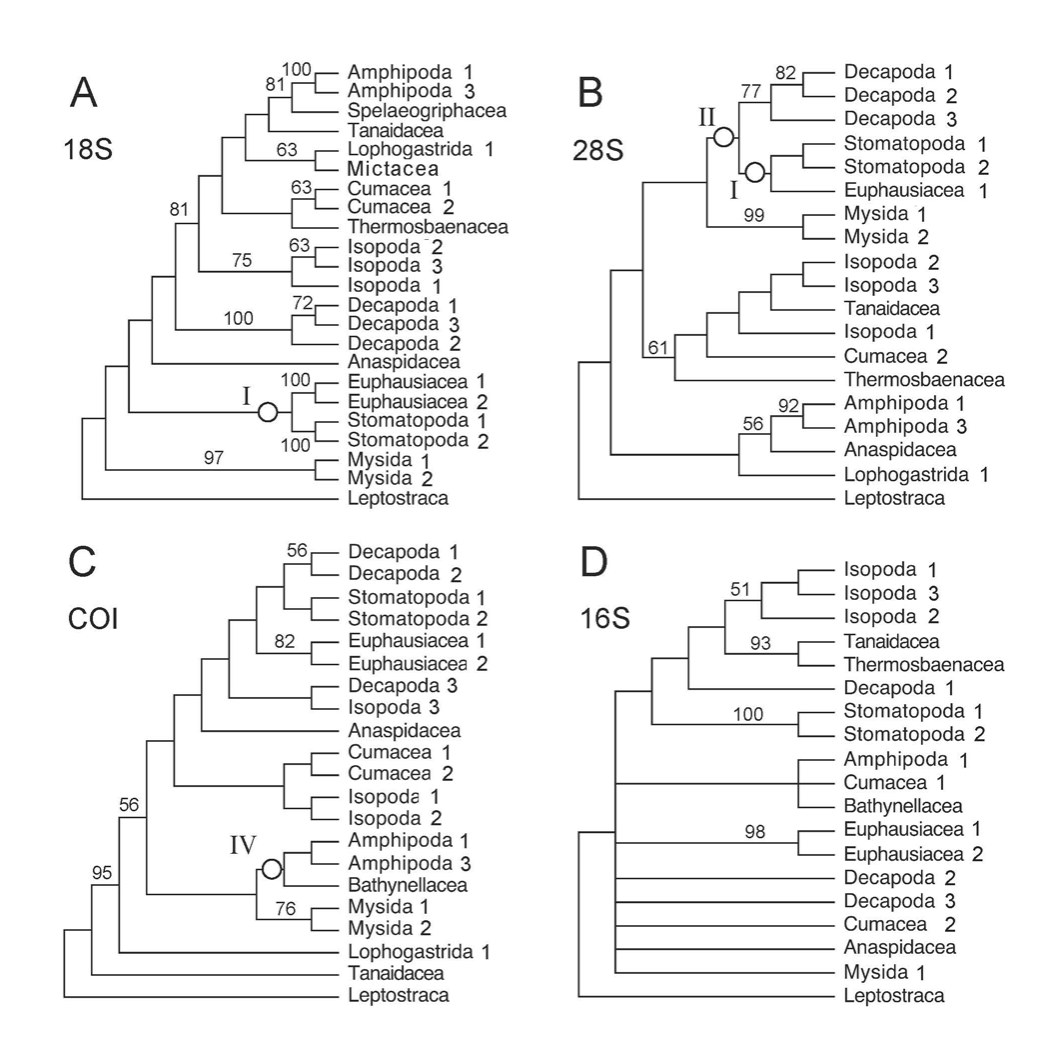
**Fitch parsimony analyses of individual partitions of molecular data**. Taxa with no informative sites for a given locus have been removed. Figures on branches indicate bootstrap percentages where these are >50%. Bootstrapping based on 1,000 resamplings, each with 1,000 random additions and TBR swapping. A. 18S rRNA: One MPT with CI = 0.50 and RI = 0.43. B. 28S rRNA: Bootstrap consensus tree, plus compatible groupings. A strict consensus of the 62 MPTs from these data (CI = 0.61 and RI = 0.49) contained only those clades with >60% bootstrap support. C. cytochrome *c *oxidase subunit I, one MPT with CI = 0.36 and RI = 0.29. D. 16S rRNA: Strict consensus of five MPTs with CI = 0.46 and RI = 0.36. Roman numerals indicate clades referred to in the text.

**Figure 3:**
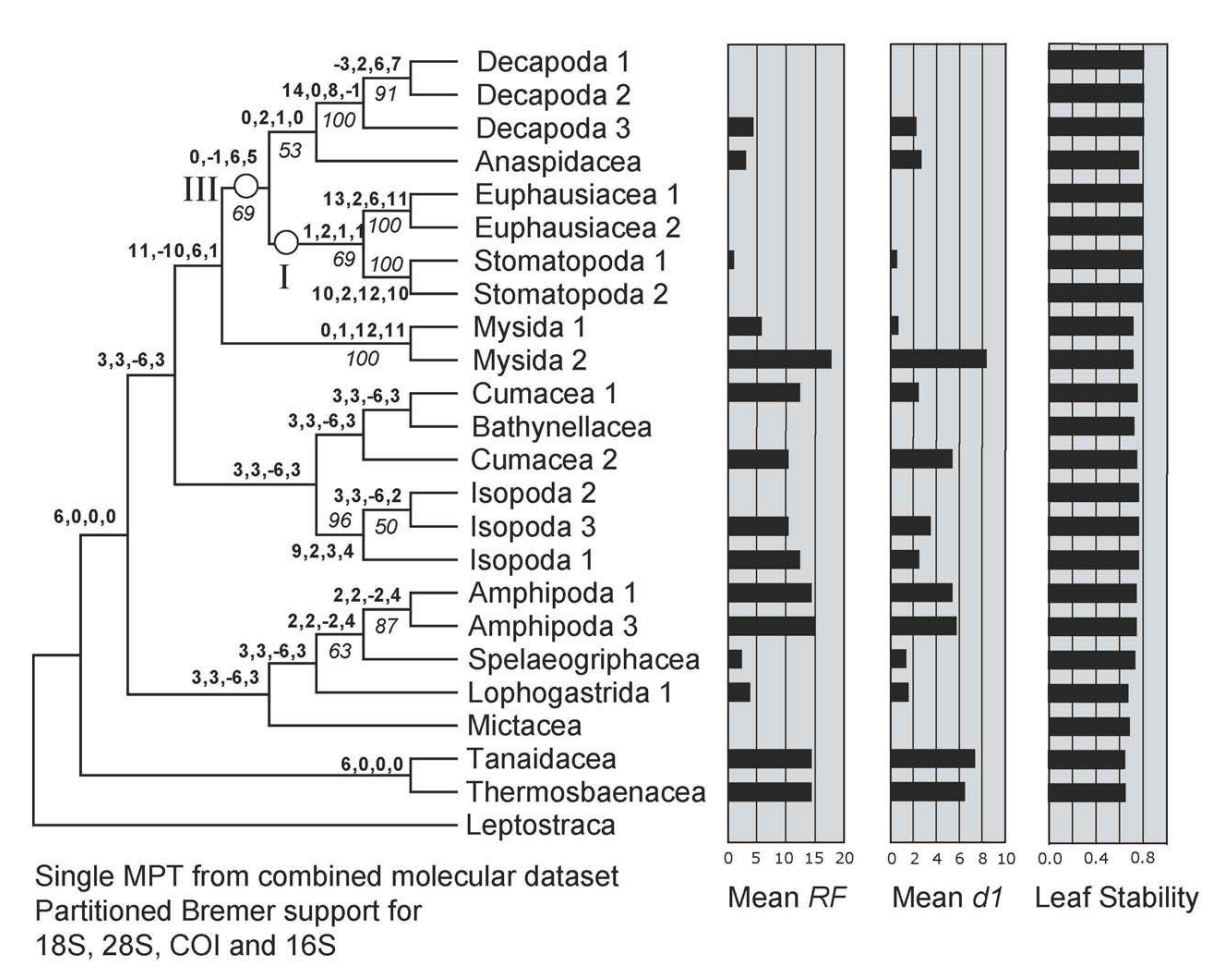
**Single MPT from Fitch parsimony analysis of combined 18S rRNA, 28S rRNA, cytochrome *c *oxidase subunit I and 16S rRNA data (CI = 0.44, RI = 0.56)**. Bold figures on branches indicate partitioned Bremer support for these four data partitions. Figures in italics indicate bootstrap support based on 2,500 resamplings, each with 1,000 random additions and TBR swapping. Values less than 50% are not reported. Histograms of mean RF and mean d1 relate respectively to the symmetrical difference distance and maximum agreement subtree distance measures of the impact upon relationships of removing individual taxa (first order jackknife). Leaf stability is calculated as the maximum value based on trees from the first 2,000 bootstrap replicates. Roman numerals indicate clades referred to in the text.

**Figure 4:**
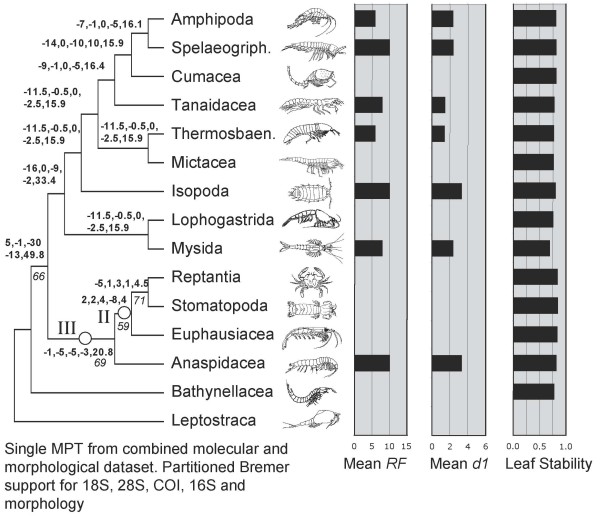
**Single MPT from Fitch parsimony analysis of combined 18S rRNA, 28S rRNA, cytochrome *c *oxidase subunit I, 16S rRNA and morphological data for a reduced set of taxa (CI = 0.50, RI = 0.29)**. Bold figures on branches indicate partitioned Bremer support for these five data partitions. Figures in italics indicate bootstrap support based on 2,500 resamplings, each with 1,000 random additions and TBR swapping. Values less than 50% are not reported. Histograms of mean RF and mean d1 relate respectively to the symmetrical difference distance and maximum agreement subtree distance measures of the impact upon relationships of removing individual taxa (first order jackknife). Leaf stability is calculated as the maximum value based on trees from the first 2,000 bootstrap replicates. Roman numerals indicate clades referred to in the text.

**Figure 5:**
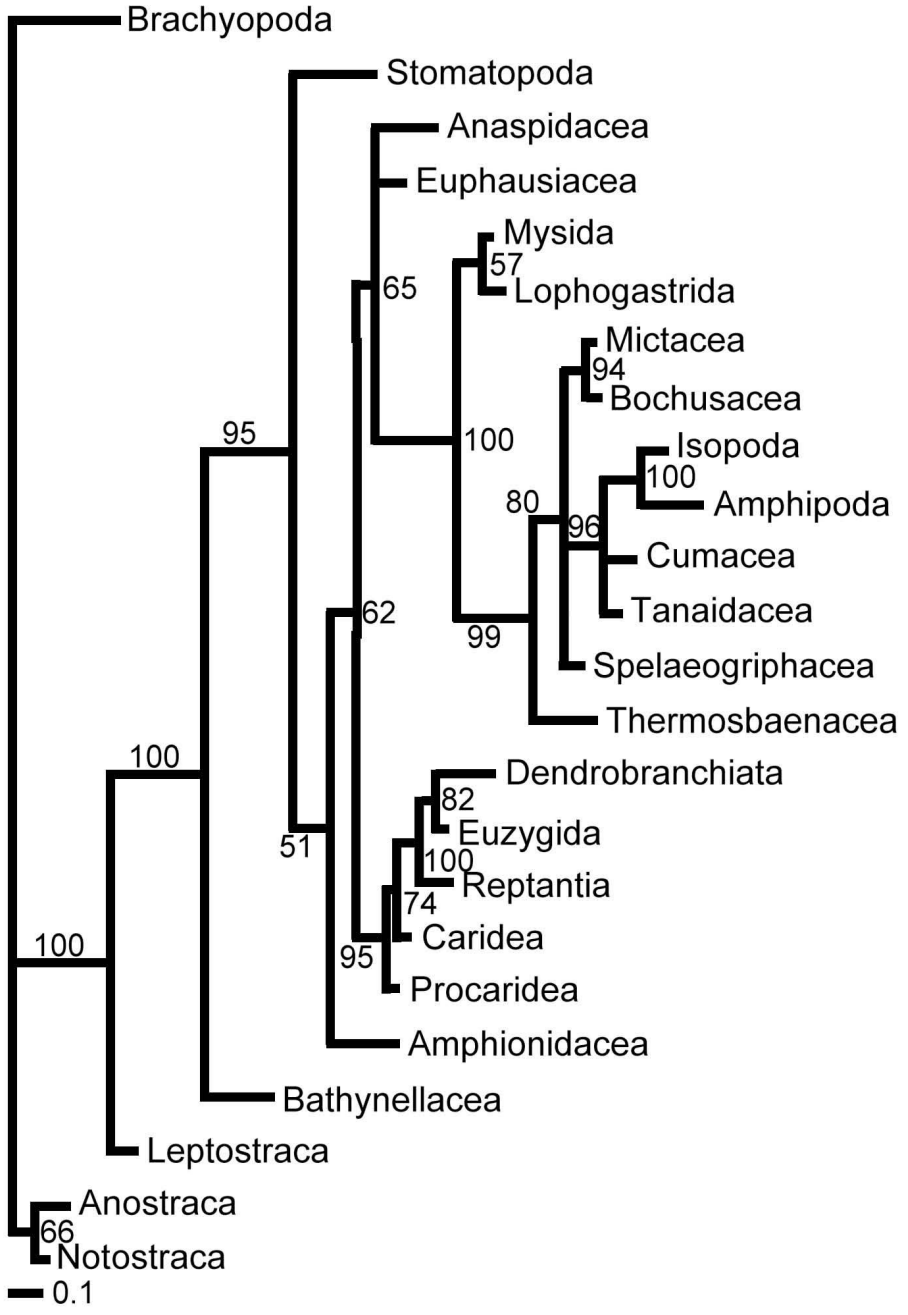
**Bayesian analysis of the morphological partition with all characters treated as non-additive. Posterior probabilities are indicated on the branches**.

**Figure 6:**
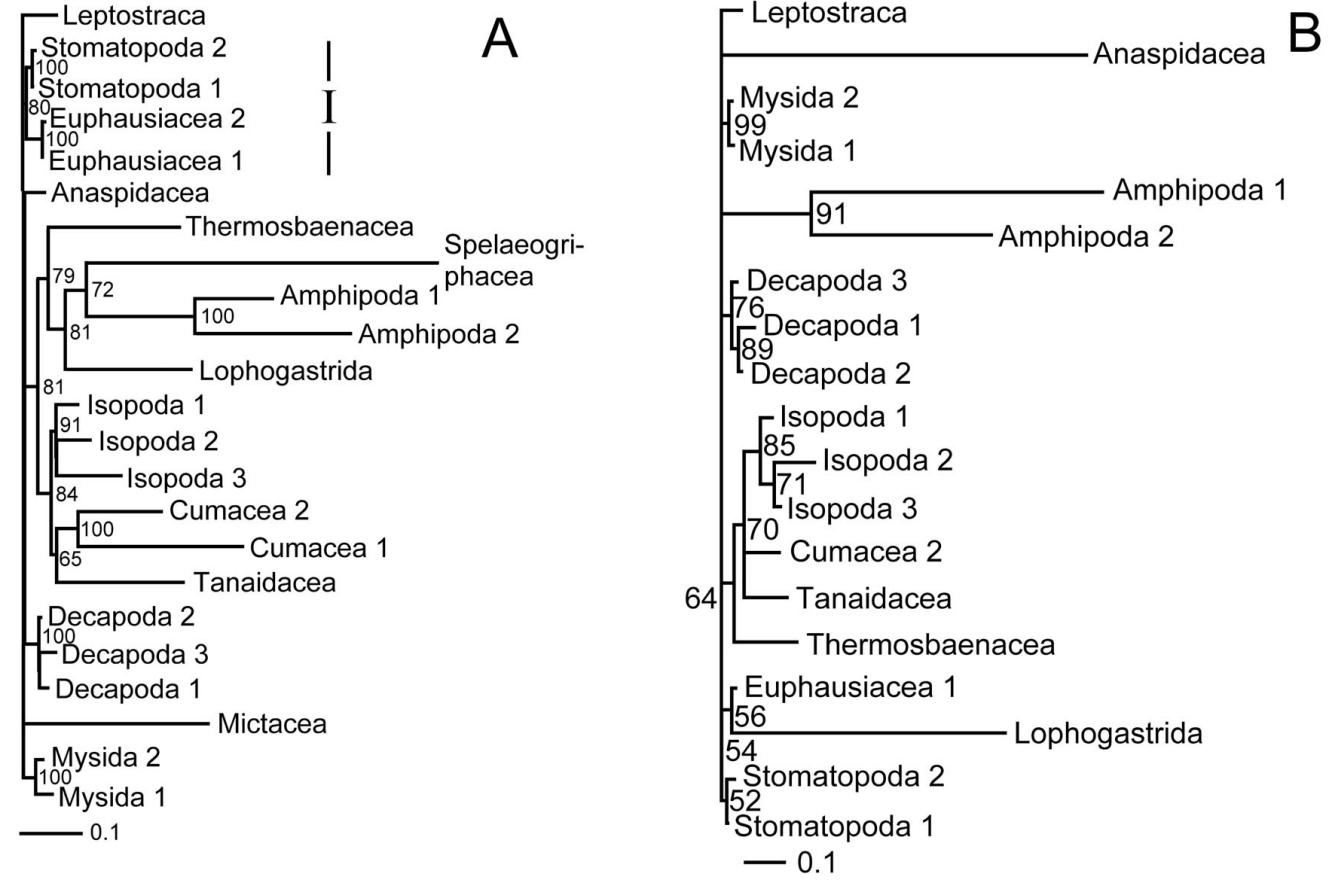
**Bayesian analyses of the 18S rRNA (A) and 28S rRNA (B) partitions**. Posterior probabilities are indicated on the branches. Roman numeral indicates a clade referred to in the text.

**Figure 7:**
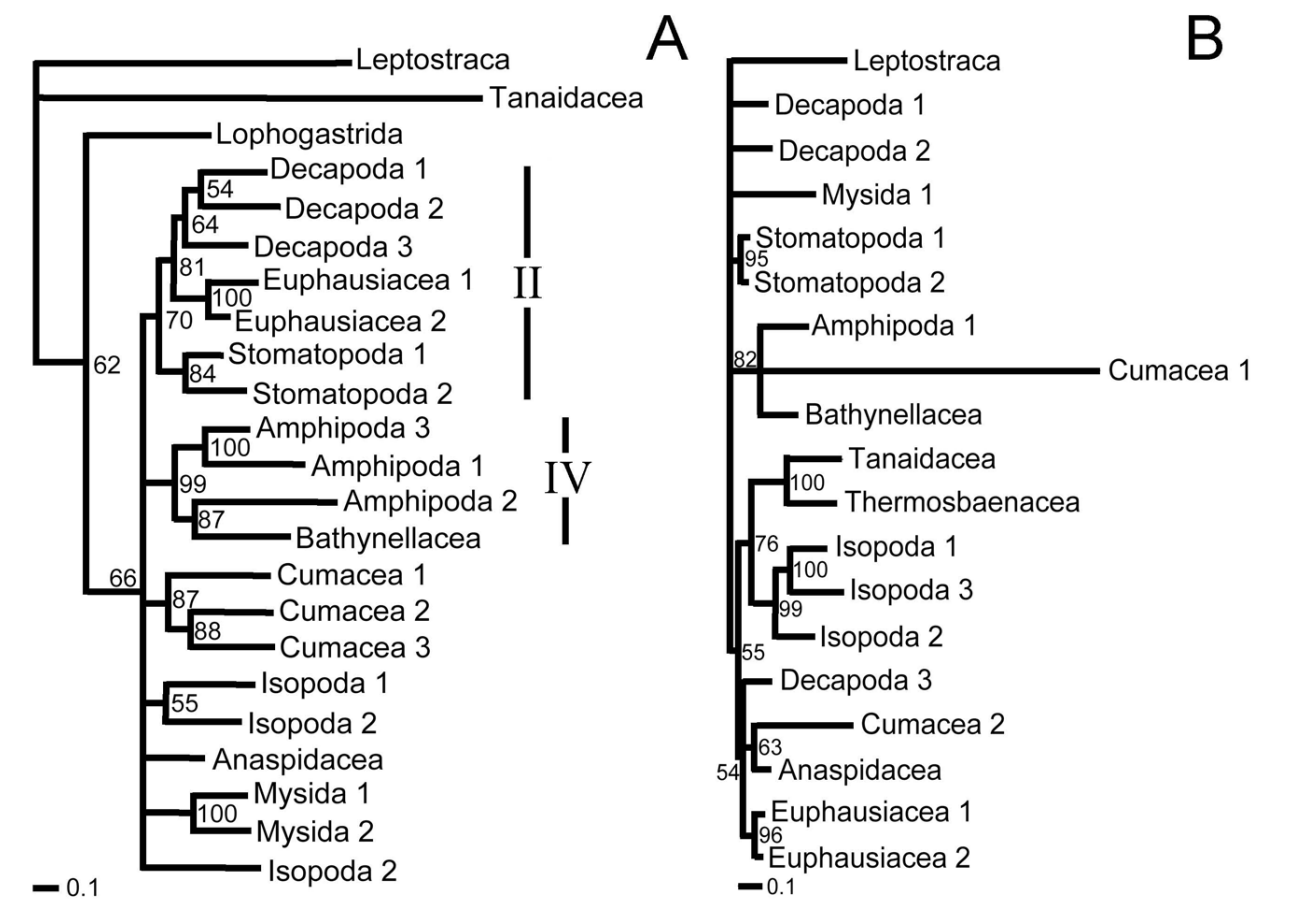
**Bayesian analyses of the mitochondrial cytochrome *c *oxidase subunit I (A) and 16S rRNA partitions**. Posterior probabilities are indicated on the branches. Roman numerals indicate clades referred to in the text.

**Figure 8:**
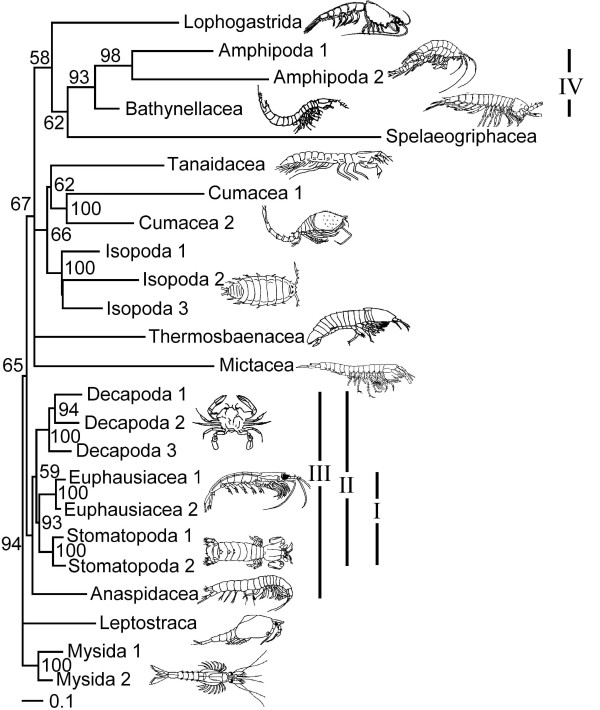
**Bayesian phylogeny of Eumalacostraca based on combined 18S rRNA, 28S rRNA, cytochrome *c *oxidase subunit I, and 16S rRNA and sequences (MOL)**. Posterior probabilities are indicated on the branches. Roman numerals indicate clades referred to in the text.

**Figure 9:**
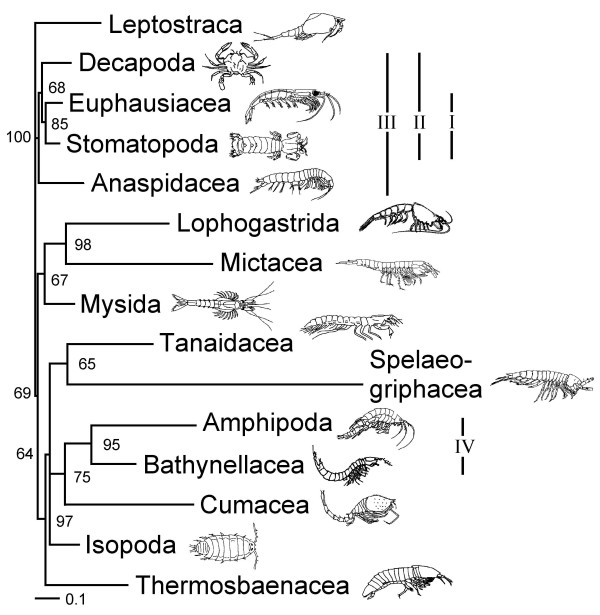
**Bayesian phylogeny of Eumalacostraca based on combined 18S rRNA, 28S rRNA, cytochrome *c *oxidase subunit I, 16S rRNA and morphological (MOLMOR) evidence**. For Amphipoda, Cumacea, Isopoda, Decapoda, Euphausiacea, Stomatopoda, and Mysida we only included the sequences with numeral '1' from Table 1. Posterior probabilities are indicated on the branches. Roman numerals indicate clades referred to in the text.

A single most parsimonious tree resulted from the analysis of the morphological characters alone (Figure 1). Trees resulting from parsimony analyses of individual molecular partitions are given in Figure 2. Combined parsimony analyses for all molecules, and molecules plus morphology, are given as Figures 3 and 4 respectively. The Bayesian morphological tree is given as Figure 5, while Bayesian trees from the individual molecular partitions are presented in Figure 6 and 7. Combined Bayesian analyses of all molecules and molecules plus morphology are given in Figures 8 and 9 respectively.

### The congruence of partitions

The results of the individual pairwise ILD tests are reported in Tables 2 and 3. ILD tests for both the combined molecular, and combined molecular and morphological data sets partitioned simultaneously into separate loci/morphology were also highly significant (P < 0.001). For the larger, 24 taxon, molecular data set, all comparisons, except that between 16S rRNA and cytochrome *c *oxidase subunit I for the ILD test, were highly significant (and the hypothesis of congruence was therefore rejected). It is probable that the 16S rRNA and cytochrome *c *oxidase subunit I comparison passes the test because the signal in one or both subsets of data is very weak (see Figures 7 and 2). The TILD tests of topological congruence confirmed this: all partitions (including 16S rRNA vs. cytochrome *c *oxidase subunit I) yielded significantly incongruent relationships (irrespective of the support for those relationships). Moreover, the PBS analyses of combined molecular and total evidence (Figures 3 and 4) pick up conflict between the signals for 16S rRNA and cytochrome *c *oxidase subunit I for several nodes. For the smaller, fifteen taxon data set including morphology, eight of the ten ILD comparisons yielded a significant result (Table 3). Comparisons showed that only the 16S rRNA and cytochrome *c *oxidase subunit I partitions, and 18S rRNA and 28S rRNA partitions passed the ILD test.

Such a finding of incongruence between morphology, mitochondrial and nuclear sequences is not uncommon [48,49], and the ILD test is known to be conservative, sometimes suggesting conflict where none exists [50]. Given the acknowledged interpretational ambiguities associated with the results of ILD and TILD tests [51] we investigated the effects of combining all of the data.

### Contribution of data partitions to combined evidence: partitioned Bremer support

PBS analysis highlighted moderate conflict between partitions for the MOL analysis. As can be seen in Figure 3, for 13 out of 21 within-ingroup-nodes, one (but never more than one) partition conflicted with the other three. In ten of these 13 cases, a mitochondrial partition (in 9 cases, cytochrome *c *oxidase subunit I) conflicted with the other partitions. In striking contrast, all 12 ingroup nodes of the MOLMOR analysis displayed conflict, as can be seen in Figure 4. For eight of these, the morphological partition conflicted with all the molecular partitions that contributed clade support. Remarkably, in all eight cases, morphology contributed positive support to the node, suggesting that the parsimony analysis of MOLMOR evidence is resolved strongly on the basis of morphology.

### Topology of Bayesian and parsimony trees

Many of the clades found in the combined evidence trees are at odds with traditional ideas based on morphological evidence, and have therefore not (yet) received names. We refrain from proposing new names for these clades because our results are equivocal. We label the clades with numbers, which are referred to in summary table 4 and the figures. The Bayesian analyses based on combined molecular (MOL), and molecular + morphological (MOLMOR) evidence (Figures 8 and 9) share a number of clades:

**Table 4:** Clades shared between Bayesian analyses of combined evidence.

Sister group hypothesis	Morphology	16S rRNA	Cyt *c *ox. Sub I	18S rRNA	28S rRNA	Combined Molecules	Total evidence
Clade I	-	-	-	+	-	1	+
Clade II	-	-	+	-	-	+	2
Clade III	-	-	-	-	-	1	2
Clade IV	-	-	+	na	na	+	+

As discussed in the text, only the Bayesian MOR and MOLMOR analyses showed significant congruence. We tabulate the shared clades (see text for clade membership), and indicate the instances where the combined evidence parsimony analyses agree.

na = not applicable because one or more taxa is not represented in this partition

+ = supports this clade

- = conflicts with this clade

^1 ^= also in parsimony MOL analysis

^2 ^= also in parsimony MOLMOR analysis

I. Euphausiacea + Stomatopoda

II. Euphausiacea + Stomatopoda + Decapoda

III. Euphausiacea + Stomatopoda + Decapoda + Anaspidacea

IV. Amphipoda + Bathynellacea

However, we stress that the Bayesian posterior probabilities were statistically insignificant (<0.95) for all clades uniting higher taxa in the MOL tree. In this tree (Figure 8) just three groups of higher taxa had posterior probabilities greater than 0.90: clade I (0.93), III (0.94), and IV (0.93). Combining morphological with the molecular data (Figure 9) raised the posterior probabilities of clade III to 1.00 and clade IV to 0.95, and additionally produced a clade containing Thermosbaenacea, Bathynellacea and the peracarid taxa with a support of 0.97.

The Bayesian MOL tree (Figure 8) and the tree based on morphological data alone (MOR) (Figure 5) were strikingly different, as was also the case for the MOL and MOR trees based on parsimony (Figures 3 and 1). The Bayesian morphological tree showed none of the clades shared between the molecular and total evidence analyses.

The parsimony tree based on the MOL data (Figure 3) shared only clades I and III with the Bayesian combined evidence trees, while the parsimony MOLMOR tree (Figure 4) only shared clades II and III with the Bayesian trees. With the exception of the sister group relationship between Amphipoda and Spelaeogriphacea, the parsimony MOL and MOLMOR trees were strikingly different. The bootstrap values for the parsimony analyses were generally very poor.

The parsimony analyses of the MOL partitions are reported in Figure 2. The parsimony and Bayesian analyses of the morphological data (Figures 5 and 1) concurred on the monophyly of Mysidacea, the sister group relationship of Amphipoda and Isopoda, and the non-monophyly of Syncarida. However, these analyses also displayed some conspicuous differences, notably in their placement of Euphausiacea, the relative positions of Mysidacea and Thermosbaenacea, and the position of Anaspidacea.

Bayesian analyses of the morphological data with or without explicit character ordering and weighting as specified in additional file 1 yielded identical trees, although with the latter slightly less resolved. Removing explicit character ordering and weighting from the parsimony analyses of the morphological dataset had modest effects. Inferred relationships were changed less than by the removal/inclusion of some terminals (i.e., Leptostraca, Euphausiacea and Amphionidacea). Weighting all characters to unity (but retaining order for those characters previously ordered) resulted in a single tree, differing from the original by d1 = 6 and RF = 12. The positions of Tanaidacea and Cumacea within the peracarids were affected, and Euphausiacea emerged as sister group to the peracarids. Additionally, relationships within the remaining eucarids were altered, with the subtree of Procaridea to Dendrobranchiata re-rooted on Dendrobranchiata (such that Procaridea + Caridea formed the most highly internested clade). Additionally treating all characters as unordered resulted in four trees, one of which was identical to that for the flat weighted but ordered analysis. The strict consensus of these four introduced just one tritomy in the peracarids and one in the outgroup. Since these changes were comparable to those resulting from slight differences in taxon sampling, we did not explore alternative ordering and weighting regimes in the combined or Bayesian analyses.

### Test of internal branch lengths

In our molecular trees, internal branches were markedly shorter than most terminal branches. Conspicuously short branches might actually represent molecular polytomies [52]. Hence, we used a likelihood ratio test to investigate whether several key branches were significantly longer than zero. For our 18S rRNA partition (Figure 6), the putative clade of Spelaeogriphacea and Amphipoda [5,6] failed this test (P < 0.01). The same held true for the clade of Tanaidacea and Cumacea, and the clade uniting all taxa except Leptostraca, Mictacea, and Mysida. While our combined molecular evidence (Figure 8) instead suggested that Bathynellacea is the sister group to Amphipoda, this was also "supported" by an internal branch length not significantly different from zero (P < 0.01). The same is true for the branch uniting both Bathynellacea and Spelaeogriphacea with Amphipoda, the clade of Tanaidacea and Cumacea, and the clade uniting all taxa except Leptostraca and Mysida.

### Relative rates of evolution and long-branch attraction

**Figure 10:**
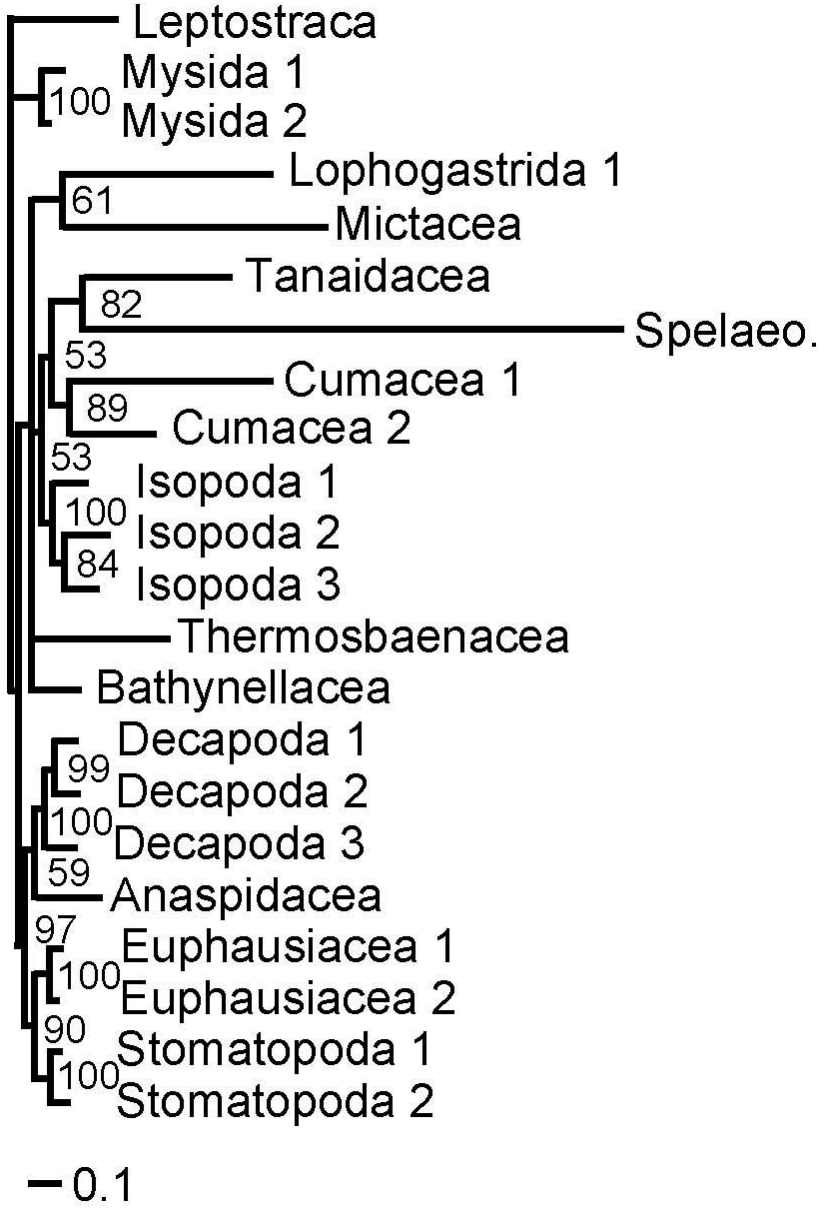
**Taxon deletion experiment**. Bayesian analysis of the combined molecular data, excluding Amphipoda. Posterior probabilities are indicated on the branches.

Long branch attraction (LBA) can result from rate heterogeneity across taxa. Likelihood ratio tests strongly rejected rate constancy in both the 18S rRNA and MOL datasets. The distance-based relative rate test confirmed this for the 18S rRNA data (P < 0.001, using a Bonferroni correction for the number of tests). Taxon deletion was then used to test for the possibility of LBA empirically. A clade of Spelaeogriphacea and Amphipoda has been recovered in previous studies [5,6], but this clade is not supported by unique morphological characters [3], and both taxa have long terminal branches. When Spelaeogriphacea was deleted from the MOL analysis the tree remained otherwise unchanged, with Amphipoda remaining the sister group to Bathynellacea. However, when Amphipoda was removed (Figure 10), the position of Spelaeogriphacea changed dramatically, becoming the sister group of Tanaidacea. This strongly suggests the possibility that Spelaeogriphacea is artificially attracted to Amphipoda, which is consistent with the zero length of the branch supporting the clade.

We stress that relative rate tests, likelihood ratio tests, taxon deletion experiments and comparisons of trees generated from different data partitions and using different methods cannot prove LBA, but they are suggestive of this explanation (or, at very least, consistent with it). None of our tests allowed us to discount the possibility of long-branch artefacts.

### The impact of taxa on inferred relationships

We also measured the impact of individual taxa upon inferred relationships in a more exhaustive way using first order jackknifing (Figures 3, 4, 1). In general terms, the two measures of impact (RF and d1) were very strongly correlated for taxa on each tree. Moreover, there was a wide range of values for the terminals on each tree, some taxa having no effect and others having a more marked impact. However, when comparing the morphological (MOR), molecular (MOL) and total evidence (MOLMOR) trees, the same taxa behaved very differently. This is consistent with our finding of conflicting signals in the morphological and molecular partitions. In the MOR dataset, eucarids had a higher impact on average than peracarids, and the most influential ingroup taxa were Euphausiacea, Amphionidacea and Lophogastrida. For the MOL data, peracarids are more influential than eucarids, although this may reflect differences in taxon sampling. Even closely related taxa (i.e., different species within the Mysida and Isopoda) can have very different impacts (Figure 3), which underlines the importance of adequate taxon sampling.

### Leaf stability

Maximum leaf stability based on bootstrapping is illustrated for the parsimony analysis of morphology, the combined molecular dataset, and the total evidence dataset (Figures 3, 4, 1). The leaf stabilities of all taxa in each of these analyses were very similar. Hence, no taxa reduce branch support spectacularly more than their neighbours. Again, the trends (insofar as these can be detected) are different for the three analyses, which reflects the different signals present.

## Discussion

This is the first study to attempt to resolve eumalacostracan phylogeny using a combination of evidence from multiple molecular loci and morphology. Our results variously confirm or reject previously proposed phylogenetic hypotheses, although it should be stressed that many conclusions remain tentative in the absence of unequivocal phylogenetic signals. The most striking findings are the relatively strong conflict between molecular and morphological evidence, and the probable effects of systematic error (including possible LBA) on these and previously published results. Although the ILD tests for the restricted 15 taxon dataset suggest that the nuclear and mitochondrial partitions are mutually conflicting, the PBS analysis instead reveals that in most cases of molecular conflict, it is the cytochrome *c *oxidase subunit I partition that conflicts with the remaining molecular partitions. However, when morphological and molecular evidence are combined, morphology conflicts strongly with all molecular partitions. Notwithstanding, the morphological signal has a strong effect on the relationships inferred in the MOLMOR tree (especially for the parsimony analysis), which differs considerably from the MOL tree. We acknowledge that such differences may also result from differences in taxon sampling. However, strong conflict between molecular and morphological evidence has been noted before for eumalacostracan relationships [3,5], although it had not been quantified in a combined analysis. Our analyses demonstrate that some of the conflict hitherto flagged up as "surprising" or "disturbing" (notably the sister group relationship between Spelaeogriphacea and Amphipoda based on 18S rRNA) dissolves because of equivocal molecular evidence and possible LBA.

We stress that the clade support measures in our analyses are generally low. Hence, while molecular and morphological conflict is *relatively *strong, neither signal is especially robust. However, given the level of interest in the conflict between molecular and morphological trees [53,54], it will be interesting to revisit the issue with new datasets in the future.

### Clade support values

In both the MOL and MOLMOR analyses, posterior probabilities and bootstrap support values were generally insignificant for nodes uniting higher-level taxa. This is unsurprising given that analyses of the separate molecular partitions also had poor support. What was surprising, however, was that in the Bayesian MOL analysis, a clade of Bathynellacea + Amphipoda had one of the three highest posterior probabilities (0.93), but was nevertheless "supported" by a branch not significantly longer than zero. This result was not apparent from branch lengths alone. The anomaly occurred because Bathynellacea was represented by mitochondrial data alone, whereas branch lengths were scaled as the expected number of changes per site. This finding highlights the need for caution when interpreting clade support measures, especially posterior probabilities that are lower than 0.95 [55].

### Branch lengths, relative rates, and LBA

Two striking features of previous molecular phylogenetic analyses [5,6,15] are the combination of short internal branches and long terminal branches, and conspicuous differences in the terminal branch lengths of ingroup taxa. In particular, non-mysid peracarid species exhibit much longer branches than the remaining taxa. Because these long-branch taxa group together, and because some of the longest branching taxa surprisingly resolve as sister groups in the absence of morphological support (Amphipoda and Spelaeogriphacea), it is possible that these relationships are affected by LBA. Spears et al. (2005) [5] noted these long branches, and concluded (p. 142) that they can generally be "viewed as an indication that a lineage either has had an ancient divergence followed by an extended period of independent evolution or has an accelerated rate of evolution." They rejected the latter possibility in favour of the former, because "a likelihood-ratio test did not find evidence of significant heterogeneity in nucleotide-substitution rates among lineages for a given topology." We question these conclusions for several reasons.

Firstly, if their likelihood-ratio test indicated that all sequences evolved according to a molecular clock, one would not anticipate pronounced differences in branch lengths between taxa. The only way a molecular tree of extant species can accommodate terminal branches of differing lengths under a clock is if internal branches are also of different but complementary lengths so as to average out apparent rates. This does not appear to be the case in Spears et al.'s phylograms. Secondly, Spears et al. preferred ancient divergence as the explanation for long branch lengths because LB taxa such as Spelaeogriphacea have a Palaeozoic fossil record. However, they also noted that equally ancient taxa may have markedly shorter branch lengths. Thirdly, our own likelihood-ratio test on the 18S rRNA data partition strongly rejected rate constancy, a finding congruent with the distance-based relative rate test. In fact, when the distance-based test is performed while not taking tree topology into account (i.e., considering every taxon as a separate lineage), Spelaeogriphacea is found to evolve at a rate significantly faster than any other included species, with the exception of the fastest evolving amphipod.

Strong rate heterogeneity and very short internal branches (indistinguishable from zero for several groupings) mean that LBA is likely to be a problem in our 18S rRNA and other data partitions. The outcome of distance-based relative rate tests has been used to exclude fast clock taxa from phylogenetic analyses to prevent LBA [44,45]. Following this advice would have made the present study impossible (at least for the 18S rRNA data). This conclusion is supported by analyses of the combined MOL and MOLMOR data sets, where exclusion of Amphipoda (the longest branch taxon after Spelaeogriphacea) caused Spelaeogriphacea to significantly change position, grouping instead with another long branch taxon (Tanaidacea). Worryingly, our relative rates tests confirm the prediction (based on branch length differences in 18S rRNA trees), that subclades have characteristic but very disparate rates. The trees of Meland & Willassen (2007) [6] illustrate this well: slow evolving mysids, euphausiaceans, and stomatopods form one clade, while the fastest evolving decapods group with a clade of fast evolving peracarids, thereby making Decapoda paraphyletic.

### Additional sequence and phylogenetic analyses: flogging a dead crab?

Although this is arguably the most methodologically thorough investigation of eumalacostracan phylogeny to date, we acknowledge that it could be refined in several ways. Among other things refinements could include additional exploratory analyses of data signal and conflict, separate analyses of stems and loops for the ribosomal genes, the use of different or simultaneous sequence alignments, incorporation of the very latest morphological evidence [56], or likelihood testing of alternative phylogenetic topologies. In the light of our results, however, we are convinced that this would be largely futile for available data. The combined phylogenetic signals in the molecular and morphological partitions are clearly insufficient to resolve a robust phylogeny. Tweaking the analyses in increasingly sophisticated ways is unlikely to change this. Instead, future time and effort should be invested in enlarging the molecular and morphological datasets. In particular, we advocate the pursuit of data from hitherto unexploited nuclear markers.

## Conclusion

Despite the combination of molecular and morphological evidence, eumalacostracan relationships remain tenuous. A consensus based on the total evidence analyses, and supported by clades with at least a posterior probability of 90 or 70% bootstrap, yields just four clades:

1. Euphausiacea + Stomatopoda

2. Reptantia (Decapoda) + Stomatopoda

3. Anaspidacea + Stomatopoda + Euphausiacea + Decapoda

4. Amphipoda + Bathynellacea

Two caveats apply. Firstly, the first and second clades are in conflict, and based on Bayesian and parsimony analysis, respectively. Secondly, the branch supporting the fourth clade is not significantly different from zero length. The third clade is the higher-level clade with the highest support to emerge from both the Bayesian and parsimony analyses of both combined molecular and total evidence analyses. It is the only Bayesian clade with statistically significant clade support (based on total evidence). The reasons for this lack of resolution include the significant conflict between morphology and molecules, the sensitivity of inferred relationships to the inclusion of morphological data, the low clade support values in both Bayesian and parsimony analyses, the very short internal branches, the heterogeneous substitution rates between taxa, the possibility of LBA, and the disagreement between Bayesian and parsimony analyses of combined MOLMOR evidence.

The fossil record indicates that Malacostraca evolved no later than the Silurian [7,57,58] and Eumalacostraca no later than the Devonian. Most major eumalacostracan lineages appear in the Carboniferous. The short internal branches separating major groups in our trees may indicate an initial, rapid radiation, consistent with the fossil record. This would make phylogenetic reconstruction especially difficult. Current evidence may be insufficient to resolve such ancient divergences: a problem that additional data may yet solve.

Higher-level metazoan phylogenetics has advanced greatly in recent years, and lessons learned in this endeavour may be applied to the eumalacostracan problem (for an overview see [59]). Both groups probably radiated rapidly, and while the Metazoa clearly did so earlier, the antiquity of the two divergences is of the same order of magnitude. Molecular phylogenies of both groups were based initially on rRNA sequences. Although these informed a greatly revised understanding of metazoan phylogeny, the limitations of relying on these loci alone have become clear too.

Workers seeking to expand the nuclear and mitochondrial dataset for Eumalacostraca must consider carefully the issue of taxon sampling. Given the potential for LBA, fast evolving species should be avoided. When included, exploratory analyses for the potential of LBA are necessary. For example, a recent study of 18S rRNA data for stygiomysids concluded that they are not related to mysids, but are rather the sister group of Mictacea [6]. However (although not included in our dataset here), a preliminary analysis indicated that stygiomysids share a high substitution rate with mictaceans, while mysids evolve significantly slower. Moreover, when Mictacea was deleted from the analysis, stygiomysids jumped to a very different position in the tree, appearing as the sister group to slow evolving decapods. This at least admits to the possibility that their phylogenetic position based on 18S rRNA evidence is an LBA artefact.

Species sampling within higher taxa should be increased as well. The results of our taxon deletion experiments (Figure 10) suggest that certain species within given higher taxa (e.g., mysids and isopods) may have disproportionate effects on tree topology. However, given the benefits and increasing ease of generating larger amounts of data [60], we believe that the most efficient strategy is to exploit new markers. Specifically, we advocate the use of nuclear protein coding genes for several reasons. Firstly, the acknowledged benefits of nuclear coding genes. There is a vast reservoir of nuclear loci evolving at a variety of rates that are able to address divergences at a range of depths. Not only can these be aligned more confidently via amino acid translation, but also amino acid sequences offer one possible way to ameliorate LBA effects that may plaque analysis of nucleotide sequences. Secondly, and more specifically, large phylogenomic data sets are based on nuclear protein coding loci. Testing the utility of these same markers for Eumalacostraca (and other lower level arthropod taxa) will foster integration of these independent subtrees via primary data, which maximizes explanatory power. When the subtrees are based on minimally overlapping or isolated datasets, by contrast, we limit ourselves to integrating them through supertree methods [61].

## Authors' contributions

RAJ designed the study, compiled the molecular dataset, performed the sequence and Bayesian analyses, tested branch lengths and relative rates, prepared figures and led the write-up. CND and MF performed preliminary data gathering and phylogenetic analyses. CND also prepared figures. MAW conceived the study, compiled the morphological dataset, performed the tests for phylogenetic signal and congruence of partitions, did the leaf stability and taxon impact analyses, performed the parsimony analyses, wrote the paper and prepared figures. All authors read and approved the final manuscript.

## Supplementary Material

Additional File 1 **Morphological character details.** List of morphological characters used in parsimony analyses with details of ordering and weighting. Click here for file

Additional File 2 **Morphological character data.** Newly synthesized data set incorporating morphological data sets from Poore (2005) [3], Richter & Scholtz (2001) [4], Wills (1998) [7], Pires (1987) [8], Wills (1997) [9] and Schram (1998) [16]. Click here for file

Additional File 3 **18S data set used in analyses of Eumalacostraca.** Nexus file of Meland & Willassen (2007) [6] 18S rRNA data. Click here for file

Additional File 4 **16S data set used in analyses of Eumalacostraca.** Nexus file of 16S rRNA data (Camacho et. al 2002) [11]. Click here for file

Additional File 5 **28S data set used in analyses of Eumalacostraca.** Nexus file of 28S rRNA data. Click here for file

Additional File 6 **COI data set used in analysis of Eumalacostraca.** Nexus file of cytochrome *c *oxidase subunit I data (Guzik et. a., 2008) [19]. Click here for file

Additional File 7 **All genes combined. Concatenated data file of all COI, 16S rRna, 28S rRna and18S rRNA data used in analyses.** Click here for file

Additional File 8 **Total evidence data file.** Combined molecular and morphological data dataset. Click here for file
