# Supplementary material for: Eumalacostracan phylogeny and total evidence: limitations of the usual suspects
Source: BMC Evol Biol. 2009 Jan 27;9:21. doi: 10.1186/1471-2148-9-21 (PMC2640363; doi:10.1186/1471-2148-9-21)
Supplement: Additional File 1 — Morphological character details. List of morphological characters used in parsimony analyses with details of ordering and weighting. [file 1471-2148-9-21-S1.pdf]

Appendix I

List of morphological characters

Sources principally Pires (1987), Wills (1997), Schram & Hof (1998), Richter & Scholtz (2001) and Poore (2005).

Cephalic shield and tergites

1. Carapace: Absent or as a simple head-shield (0). Present (1). It is debatable whether the condition in the Mictacea should be scored as a head shield or a carapace. Schram (1986) and Wills (1997) referred to the structure as a head-shield, despite the presence of marginal flaps that partially envelop the bases of the cephalic appendages. Richter and Scholtz (2001) preferred to code the structure as a carapace. We adopt the latter approach here.

2. Posterior extent of carapace: Well-developed, covering the thorax (0). Short, covering only the anteriormost thoracic segments (1).

3. Dorsal fold on adult: Absent (0). Present (1). A fold arising from and attached to a thoracic segment. In Malacostraca, this always arises from the posterior margin of the cephalothoracic shield (Dahl 1991).

4. Branchiostegal flaps: Absent (0). Present (1).

5. Cephalic pleural fold: Absent (0). Present (1).

6. Carapace with respiratory function: Non-respiratory (0). Respiratory (1).

7. Ventral extent of carapace: Normal (0). All-enveloping (1).

8. Number of thoracomeres involved in forming the cephalothorax: None (0). One (1). Two (2). Three (3). Eight (4). Wills (1998) coded a similar character for the number of trunk somites giving rise to branchiostegal folds. Here, we adopt the interpretation and coding of Richter and Scholtz (2001), such that taxa without a carapace can also be coded.

9. Articulation of tergites: With no overlap (0). With overlapping pleurae (1).

10. Pleura of the second pleon segment: Pleura not overlapping that of the anterior (first) pleomere (0). Pleura overlapping that of the anterior (first) pleomere (1). Richter & Scholtz (2001)

11. Rostrum: Absent (0). Fixed (1). Articulating (2).

12. Cervical groove (just posterior of the maxillae): Absent (0). Present (1). (see also Poore 2005)

13. Cephalic kinesis / protocephalon: Absent (0). Present (1).

14. Males (at least) with transverse suture in cephalon, immediately behind the mandibles: Absent (0). Present (1).

15. Cephalic doublure: Absent (0). Present (1).

16. Carapace adductor muscles: Present (0). Absent (1).

Eyes and frontal organs

17. Compound eyes: Absent (0). Present (1).

18. Form of compound eyes: Sessile (0). Stalked (1). Lobed (2).

19. Ocular lobe: Absent (0). Present (1). An ocular lobe can be present in the absence of compound eyes, and vice versa.

20. Ultrastructure of ommatidia: Crystalline cone tetrapartite (0). Crystalline cone bipartite (1).

21. Ultrastructure of ommatidia: Crystalline cone completely round in transverse section, cone without any extensions (0). Cone with two lateral extensions (in transverse section button-like), formed by one cone cell each (1).

22. Ultrastructure of ommatidia: Crystalline cones with four cone cell processes (0). Only the two accessory cone cell processes are present; the processes of the main cone cells are missing (1). All cone cell processes missing (2).

23. Ultrastructure of ommatidia: All four cone cell nuclei lying in one plane on top of the cone (0). Nuclei of the accessory cone cells distally displaced (1).

24. Ultrastructure of ommatidia: No clear zone between crystalline cone and rhabdom (apposition eye) (0). Clear zone formed by retinular cells and/or distal pigment cells, cone and rhabdom not in direct contact (superposition eye) (1).

25. Naupliar eye sensu stricto: Absent (0). Present (1).

26. Dorsal frontal organ: Absent (0). Present (1).

27. Ventral frontal organ: Absent (0). Present (1).

28. Dorsal, nuchal or neck organ: Absent (0). Present (1).

29. Bec oculair: Absent (0). Present (1).

Antennule

30. Number of podomeres in outer ramus (exopod): 10 or more (0). 9 to 4 (1). 3 to 1 (2). Outer ramus absent (3).

31. Exopod scale-like: No (0). Yes (1).

32. Number of podomeres in inner ramus (endopod): 10 or more (0). 9 to 1 (1).

33. Statocyst in basal segment of first antenna: Absent (0). Present (1).

Antenna

34. Number of podomeres in outer ramus (exopod): None (0). 1 (1). 2-4 (2). 18 or more (3).

35. Antennal exopod modified as scaphocerite: Not modified (0). Modified (1).

36. Antennal scale (scaphocerite): As long or longer than peduncle articles 3+4 (0). Half the length of peduncle articles 3+4 (1).

37. Number of podomeres in inner ramus (endopod): 1-2 (0). 3 (1). 5 (2). 8 or more (3).

38. Antennal gland: Absent (0). Present (1).

39. Antennal naupliar process: Absent (0). Present (1)

Mandible and mandibular region

40. Number of podomeres in endopod: None (endopod absent) (0). 1-2 (1). 3-4 (2).

41. Mandibular palp: With lateral setae on articles 2 and 3 (0). With distal setae on article 3 only (1).

42. Mandibular incisor: Stout and tooth-like (0). Thin and blade-like or rudimentary (1). Absent (2).

43. Mandible with marked molar process: Absent (0). Present (1).

44. Arrangement of molar and incisor elements: Short and compact, incisor and molar closely set (0). Long, incisor and molar widely-spaced (1). Scored as inapplicable for taxa lacking an incisor, molar or both.

45. Lacinia mobilis on the adult mandible: Absent (0). Present (1).

46. Lacinia mobilis on the larval mandible: Absent (0). Present (1).

47. Paragnaths: Absent (0). Present (1).

48. Labrum: Moderate (small to medium) (0). Enlarged to extend well posterior of the mouth field (massive) (1).

49. Posterior tooth on labrum: Absent (0). Present (1).

50. Labrum expression in larva: Moderate (0). Enlarged to extend well posterior of the mouth field (1).

51. Epistome: Absent or vestigial (0). Well-developed (1).

First maxilla

52. Number of podomeres in exopod: None (exopod absent) (0). 1-2 (1).

53. Number of podomeres in endopod: None (endopod absent) (0). 1-2 (1). 3 (2).

Second maxilla

54. Endites: 8-6 in number (0). 5-4 in number (1). 3-1 in number (2). Absent (3).

55. Basal endites: Longer than wide (0). About as wide as long (1).

56. Number of podomeres in exopod: None (exopod absent) (0). 1 (1). 2 (2).

57. Exopod modified as a scaphognathite: Not modified (0). Modified (1).

58. Number of podomeres in endopod: None (endopod absent) (0). 1-2 (1). 6 (2).

59. Maxillary glands: Absent (0). Present (1).

Appendage of sixth segment

60. Protopodal endites: Present (0). Absent (1).

61. Number of podomeres in exopod: None (exopod absent) (0). 1 (1). 2-4 (2). Very numerous (3).

62. Exopod: Linear (0). With expanded basal section (1).

63. “Caridean lobe” on exopod: Absent (0). Present (1).

64. Number of podomeres in endopod: 1-2 (0). 3 (1). 4 (2). 5 (3). 6 (4).

65. Number of epipodites: None (0). One (1). Two (2).

66. Role of epipodites of first thoracopod in respiration: Respiratory and similar to those of succeeding thoracopods (0). Epipodites producing a respiratory current (irrespective of whether or not the epipodite is respiratory itself) (1). Epipodites not producing a respiratory current (and epipodite not respiratory) (2).

67. Expansion of epipodite: Short, linear (or in Isopoda, not expanded into branchial cavity) (0). Expanded into branchial cavity (1).

68. Form of epipodite: Not modified (0). Modified as a cup or spoon-shaped (respiratory) structure (1).

Appendage of seventh, eighth and ninth segments

69/78/87. Protopodal endites: Present (0). Absent (1).

70/79/88. Number of podomeres in exopod: None (exopod absent) (0). 1 (1). 2 (2). 3-4 (3). Very numerous (4).

71/80/89. Form of exopod: Flagelliform, or otherwise developed as an elongate process (0). Developed as a broad, lamelliform paddle or blade, or otherwise non-flagelliform (1).

72/81/90. Number of podomeres in endopod: 1 (0). 2-3 (1). 4 (2). 5 (3). 6 (4).

73/82/91. Number of epipodites: None (0). One (1). Two (2).

74/83/92. Oöstegites: Absent (0). Present (1).

75/84/93. Podobranch gills: Absent (0). Present (1).

76/85/94. Arthrobranch gills: Absent (0). Present (1).

77/86/95. Pleurobranch gills: Absent (0). Present (1).

General morphology of thoracic appendages

96. Position of epipodites on thoracopods 2-8: Lateral (0). At least one branch carried under the thorax (1). This character is scored as inapplicable for taxa without epipodites.

97. Posterior extent of oöstegites: As far back as thoracopod 8 (0). As far back as thoracopod 6 or 7 (1). This character is scored as inapplicable for taxa without oöstegites.

98. Reduction of the oöstegites after each brood: Oöstegites are not reduced (0). Oöstegites are reduced (1).

99. Thoracopod thorax-coxa articulation: Transverse hinge (0). Anterioposterior articulation (1). Immobile (2).

100. Thoracopods coxa-basal articulation: Dicondylic along anteroposterior axis (0). Monocondylic (1). Not articulated, or coxa and basis otherwise fused (2).

101. Thoracopods intrabasal articulation: Absent (0). Present (1).

102. Thoracopods 4-5: Achelate (0). Chelate (1).

103. Thoracopod 5-7, pedunculate setae: Absent (0). Present (1).

104. Thoracopods 5 & 6 (appendages 10 & 11): Exopod present (0). Absent (1). Stomatopods bear exopods on the 6th thoracopods, but not on the 5th (fifth maxillipedes). They are therefore scored as (1,2).

105. Thoracopod 5 (pereopod 4) exopod of female: With two or more articles (0). With one article (1).

106. Thoracopod 6 (pereopod 5) exopod: With two or more articles (0). With one article (1).

107. Thoracopod 6: Achelate (0). Chelate (1).

108. Thoracopod 7 (pereopod 6) exopod: Of two or more articles (0). Of one article (1).

109. Thoracopod 8 (pereopod 7) exopod: Present (0). Absent (1).

110. Attitude of trunk appendages relative to body: Pendant (0). Laterally displaced (1).

111. Thoracic coxal plates: Absent (0). Present (1).

112. Oöstegites with marginal setae: Present (0). Absent (1).

113. Pereopods: With a few short setae on articles (0). With a row of long setae on all articles (1).

Abdominal appendages

114. Abdomial limbs: All present (0). Posterior limbs reduced or absent (1).

115. Number of podomeres in exopod of second abdominal appendage/pleopod: None (0). One or vestigial (1). Two (2). Annulate (3).

116. Number of podomeres in endopod of second abdominal appendage/pleopod: None (0). One or vestigial (1). Two (2). Annulate (3).

117. Abdominal appendages (pleopods) modified for brooding eggs: Unmodified (0). Modified (1).

118. Gills (as a distinct filamentory or platelike structures) on abdomen: Absence (0). Present (1).

Posteriormost appendages

119. Last pleopods: Small, far from the telson, and not forming a tail fan (0). Modified to broad uropods, forming a tail fan with the telson (1). Oriented posteriorly and close to the telson, but not forming a tail fan with it (2).

120. Number of podomeres in inner rami of uropods: One (0). Two or more (1).

121. Number of podomeres in outer rami of uropods: None (0). One (1). Two or more (2).

122. Uropod numbers: None (0). One set (1). Three sets (2).

Telson and furca

123. Gross form of telson: Round, segment-like (0). Dorsoventrally-flattened (1).

124. Telson appendages (furca): Absent (0). Present (1). Richter and Scholtz (2001) score telson appendages in the Leptostraca and Bathynellacea. Schram (1986) additionally records their presence in the Euphausiacea.

Tagmosis

125. Number of maxillipeds: None (0). One (1). Two (2). Three or more (3). Richter and Scholtz (2001) prefer to break the single character for the number of maxillipeds used by Schram and Hof (1998) and Wills (1997) down into two. The first codes for the first maxilliped (presence/absence) since this is regarded as a robust homology. The second codes for the transformation of additional and subsequent thoracopods (none, second and third, or five), where homologies between groups are less certain. We assume that this second character is unordered, recognizing only that different, homologous conditions exist, but not associating these in an ordered sequence according to actual number. If so, the treatment here is different, and the entire character is treated as ordered.

126. Number of post-maxillary body segments, *including* the telson or anal somite: 14-15 (0). 16 (1). 20 or more (2).

127. Abdomen (excluding the telson/terminal division): > 7 segments (0). 6 segments (1). 5 segments (2). 4 segments (3).

128. Pleomere size: First pleomere fully developed, of similar size and appearance to the more posterior pleomeres (0). First pleomere reduced, smaller than the second pleomere (1). More pleomeres reduced (2).

129. Reduction or absence of pleon appendages: All pleon appendages present (0). All pleon appendages absent, or up to three highly-reduced and vestigial appendages clearly demarcated from those of the thorax (1).

130. Fusion of telson to the pleonite: Not fused (0). Fused (1).

Internal organs

131. Foregut dorsal caeca: Absent (0). Present (1).

132. Foregut shape: Anterior section of similar size to posterior (0). Anterior section enlarged with respect to posterior (1).

133. Foregut dorsolateral and midventral ridges: With setae (0). With teeth or ossicles (1).

134. Lateralia and inferolateralia anteriores (lateral invaginations) in the cardiac chamber: Absent (0). Present (1).

135. Superomedianum (unpaired): Absent (0). Present (1).

136. Inferomedianum anterius (midventral cardic ridge): Absent (0). Present (1).

137. Inferomedianum posterius (midventral pyloric ridge): Absent (0). Present (1).

138. Atrium between the inferomediana connecting the cardiac primary filter grooves with the pyloric filter grooves: Absent (0). Present (1).

139. Number of secondary filter grooves in the inferomedianum posterius: Numerous (0). Eight to six (1). Three (2). Two (3). One (4). Scored as inapplicable for taxa lacking an inferomedianum posterius.

140. Formation of the midgut: By ectoderm (0). At the border between the stomodaeum **a**nd proctodaeum (1).

141. Entoderm: Unpaired entoderm plates (0). Paired entoderm plates (1).

142. Trunk gut diverticulae and/or caecae: Absent (0). Present (1).

143. Position of the anus: Terminal (0). Ventral (1).

144. Position of the heart: In whole thorax and pleon (0). In thorax (1). Only in posterior part of the thorax and pleon (2).

145. Gross morphology of the heart: Elongate (0). Short and bulbous (1). (Watling, 1983).

146. Number of pairs of ostia in heart: More than five (0). Five (1). Three (2). Two (3). One (4). None (5).

147. Arteria subneuralis/supraneuralis: Absent (0). Present (1).

148. Aorta descendens (sternal artery) as the only connection between the heart and the **a**rteria subneuralis/supraneuralis: Absent (0). Present (1). Coded as inapplicable in taxa lacking **a**n arteria subneuralis/supraneuralis.

149. Aorta descendens: The undivided sternal artery passes through the ventral nervous system (0). Sternal artery branches off into three branches dorsal to the ventral nervous system, **a**ll branches passing separately through the nerve cord (1). Coded as inapplicable in taxa lacking an arteria subneuralis/supraneuralis.

150. Segmental arteries: Absent, arteries arising only from the anterior and posterior ends of the heart (0). Present (1).

151. Pleon musculature: Simple (0). Precaridoid (1). Caridoid (2).

152. Tail fan escape reaction: Absent (0). Present (1).

153. CNS: Ventral nerve cord with unfused, paired ganglia and double ventral commisures (0). Ventral nerve cord with fused ganglia (1).

154. Globuli cell clusters in the deutocerebrum asscociated with the olfactory lobe: One (0). Two (1).

Reproduction and development

155. Male gonopore location (post-maxillary trunk segment numbers): Segments 6-8 (0). Segment 11 (1).

156. First and/or second pleopods modified for sperm transfer in males: No modification or rudimentary modifications (0). Stomatopod petasma, including modifications of the exopod of the second pleopod (1). Endopod of the first pleopod completely modified for sperm transfer, modifications different in the second endopod (2).

157. Appendices internae: Absent (0). Present (1).

158. Development: Anamorphic (0). Metamorphic (1). Epimorphic or direct (2).

159. Free living larva: Present (0). Absent (1). Coded as inapplicable for taxa with epimorphic or direct development.

160. Orthonauplius: None (0). Egg nauplius only (1). Present without fronto-lateral horns (2).

161. Manca stage: Absent (0). Present (1).

162. Brood care: None (0). Brood care with thoracopods, but without feeding by the mother (1). Brood care attaching the eggs to the pleopods (2). Brood care using a dorsal brood pouch (3).

Brood care using a marsupium formed by oöstegites (4). Brood care using elongated first pleopod (5).

163. Development of appendages: Advanced development of anterior head appendages (0). Continuous anterioposterior decrease in the degree of appendage formation (1).

164. Cleavage: Superficial cleavage (0). Mixed cleavage (1). Total cleavage (2).

165. Number of ectoteloblasts: Nineteen (0). Variable (1). None (2).

166. Arrangement of ectoteloblasts: Forming a ring around the caudal papilla giving rise to embryonic ventral and dorsal material (0). Forming a transverse row (only the ventral side of the embryo is formed by ectoteloblasts and the dorsal side is closed much later in development) (1).

167. Early embryo (nauplius larva): Ventrally folded (0). With a dorsal fold (1).

168. Yolk distribution in the embryo: Posterior part of the embryo contains no yolk (0). Posterior part of the embryo contains yolk (1).

169. Number of pairs of thoracic appendages in the hatchling: Eight (0). Seven (1). Six (2). Scored as inapplicable for taxa without direct development.

170. Embryonic dorsal organ: Present (0). Absent (1).

171. Embryonic dorsal organ: Simple layer (0). Cup shaped (1).

172. Transient paired lateral organs: Absent (0). Present (1).

Sperm

173. Sperm acrosome: Present (0). Absent (1).

174. Sperm filamentous arms: None (0). Present (1).

175. Sperm nuclear membrane: Present (0). Absent (chromatin diffuse) (1).

176. Spermatophore: None (0). Present (1).

177. Sperm centriole: Present (0). Doublet (1). Centriolar root homologue (cross-striated pseudoflagellum). (2). Absent (3).

Assumptions

All characters unordered, except:

Characters with more than two states treated as ordered:
8, 30, 34, 37, 40, 53, 54, 56, 58, 61, 64, 65, 70, 72, 73, 79, 81, 82, 88, 90, 91, 115, 116, 121, 122, 125, 126, 127, 128, 146, 151, 169

All characters unweighted, except:
Characters with more than two states scaled to unit weight (ranged):

30, 34, 37, 40, 53, 54, 56, 58, 61, 64, 65, 70, 72, 73, 79, 81, 82, 88, 90, 91, 115, 116, 121, 146

References

Dahl E: **Crustacea, Phyllopoda and Malacostraca: a reappraisal of cephalic and thoracic shield and fold systems and their evolutionary significance**. *Phil Trans R Soc Lond B* 1991, **334**: 1-26.

Pires AMS: ***Potiicoara brasiliensis*: a new genus and species of Spelaeogriphacea (Crustacea, Peracarida) from Brazil with a phylogenetic analysis of the Peracarida**. *J Nat Hist* 1987, **21**:225-238.

Poore GCB: **Peracarida: monophyly, relationships and evolutionary success**. *Nauplius* 2005, **13**:1-27.

Richter S, Scholtz G: **Phylogenetic analysis of the Malacostraca (Crustacea)**. *J Zool Syst Evol Res Evol* 2001, **39**:113-136.

Schram FR: *Crustacea*. Oxford: Oxford University Press; 1986.

Schram FR, Hof CHJ: **Fossils and interrelationships of major crustacean groups**. In: *Arthropod fossils and phylogeny*. Edited by Edgecombe GD. New York: Columbia University Press; 1998: 233-302.

Watling LE: **Peracaridan dis-unity and its bearing on eumalacostracan phylogeny with a reclassification of eumalacostracan superorders**. In *Crustacean Phylogeny*. Edited by Schram FR. *Crustacean Issues* 1983, **1**:213-228.

Wills MA: **A phylogeny of recent and fossil Crustacea derived from morphological characters**. In *Arthropod relationships*. Edited by Fortey RA, Thomas RH, vol. The Systematics Association Special Volume Series 55. London: Chapman & Hall; 1997:189-209.
